# Protein arginine *N*-methyltransferase 2 plays a noncatalytic role in the histone methylation activity of PRMT1

**DOI:** 10.1016/j.jbc.2023.105360

**Published:** 2023-10-19

**Authors:** Michael J. Rowley, Riley A. Prout-Holm, Rui Wen Liu, Thordur Hendrickson-Rebizant, Olufola O. Ige, Ted M. Lakowski, Adam Frankel

**Affiliations:** 1 Faculty of Pharmaceutical Sciences, The University of British Columbia, Vancouver, British Columbia, Canada; 2 College of Pharmacy, University of Manitoba, Winnipeg, Manitoba, Canada

**Keywords:** protein arginine *N*-methyltransferase 2, histone methylation, histone H2A, differential scanning fluorimetry, mononucleosomes, epigenetics

## Abstract

Protein arginine *N*-methyltransferases are a family of epigenetic enzymes responsible for monomethylation or dimethylation of arginine residues on histones. Dysregulation of protein arginine *N*-methyltransferase activity can lead to aberrant gene expression and cancer. Recent studies have shown that PRMT2 expression and histone H3 methylation at arginine 8 are correlated with disease severity in glioblastoma multiforme, hepatocellular carcinoma, and renal cell carcinoma. In this study, we explore a noncatalytic mechanistic role for PRMT2 in histone methylation by investigating interactions between PRMT2, histone peptides and proteins, and other PRMTs using analytical and enzymatic approaches. We quantify interactions between PRMT2, peptide ligands, and PRMT1 in a cofactor- and domain-dependent manner using differential scanning fluorimetry. We found that PRMT2 modulates the substrate specificity of PRMT1. Using calf thymus histones as substrates, we saw that a 10-fold excess of PRMT2 promotes PRMT1 methylation of both histone H4 and histone H2A. We found equimolar or a 10-fold excess of PRMT2 to PRMT1 can improve the catalytic efficiency of PRMT1 towards individual histone substrates H2A, H3, and H4. We further evaluated the effects of PRMT2 towards PRMT1 on unmodified histone octamers and mononucleosomes and found marginal PRMT1 activity improvements in histone octamers but significantly greater methylation of mononucleosomes in the presence of 10-fold excess of PRMT2. This work reveals the ability of PRMT2 to serve a noncatalytic role through its SH3 domain in driving site-specific histone methylation marks.

The histone code is comprised of a multitude of posttranslational modifications on histone proteins that control access to the genetic code and determine cell fate (1). Epigenetic marks constituting the histone code are deposited by a plethora of protein-modifying enzymes, often referred to as the writers of the epigenetic code (2, 3). These marks alter the biochemical properties of histone proteins and require specific reader proteins to allow transcriptional machinery to access the underlying genes (1). Aberrant expression of epigenetic writers can lead to increased expression of oncogenic genes and repression of tumor-suppressor genes that can contribute to the onset of cancer (3, 4). Understanding the writers of the epigenetic code is of the utmost importance for developing targeted cancer therapeutics to prevent uncontrollable cell growth (4).

Among the epigenetic writers within the protein arginine *N*-methyltransferase (PRMT) family of enzymes (5, 6, 7), PRMT2 remains somewhat enigmatic. We and others have shown that it is able to catalyze the formation of monomethylarginine (MMA) and asymmetric dimethylarginine (aDMA) on histone H4, as well as on glycine- and arginine-rich and serine- and arginine-rich substrates using SAM as the methyl donor, which gets converted into SAH after methyl transfer (8, 9). However, PRMT2 is distinct from enzymes that form either the same products (PRMT1, PRMT3, coactivator-associated arginine methyltransferase 1(CARM1), PRMT6, and PRMT8), MMA only (PRMT7), or MMA and symmetric dimethylarginine (sDMA) (PRMT5 and PRMT9), in that it exhibits the lowest catalytic efficiency for methyl transfer (10). It is conceivable that we have yet to identify substrates upon which human PRMT2 exhibits high activity. Despite such low *in vitro* activity, paradoxically PRMT2 was shown to facilitate aDMA formation on histone H3 at arginine 8 (H3R8me2a) at promoter sites for transcriptional activation during *Xenopus* development (11). It was later demonstrated that PRMT2 overexpression and the H3R8me2a mark were linked to oncogenic transcriptional programming in glioblastoma multiforme, hepatocellular carcinoma, and renal cell carcinoma, in each case strongly correlating with disease severity (12, 13, 14). In this regard, PRMT2 joins the PRMT pantheon of other well-known writers with oncogenic capacity (15, 16). These other writers include transcriptional activators PRMT1 responsible for H4R3me2a, CARM1 responsible for H3R17me2a, H3R26me2a, and H3R42me2a (17, 18, 19, 20), transcriptional repressors PRMT5 responsible for H2AR3me2s, H3R8me2s, and H4R3me2s, and PRMT6 responsible for H3R2me2a (21, 22, 23, 24, 25).

Like other PRMTs, the PRMT2 monomer possesses a SAM-binding Rossmann fold, a peptide-binding groove, and dimerization arm, and it forms a toroidal shape as a homodimer comprised of catalytic cores (PDB ID: 5FUL) (9, 26). Unlike other PRMTs, PRMT2 contains an N-terminal Src-homology 3 (SH3) domain whose solution structure was solved by NMR in 2005 (PDB ID: 1XP2). The AlphaFold full-length structure of PRMT2 shows the SH3 domain attached to the rest of PRMT2 through a highly flexible linker (Fig. S1). This auxiliary SH3 domain has been shown to bind polyproline stretches on proteins involved in splicing and cell scaffolding (27, 28, 29). The importance of the SH3 domain for PRMT2 function was initially highlighted in a study showing that its removal resulted in an interaction loss between the methylation substrate hnRNP E1B-AP5 and its ostensible methyltransferase PRMT2 (HRMT1L1) in cultured H1299 cells (30). In activity assays, removal of the SH3 domain was shown to further reduce the already low catalytic activity of PRMT2 (8, 9). While not linked to enzymatic activity per se, full-length PRMT2 has been shown to function as a transcriptional coactivator of several nuclear receptors (31), as well as influence functions of IκB-α (32), and the retinoblastoma gene product (33). It is unclear with our present understanding of PRMT2 if it functions in cells as an active methyltransferase, as a structural subunit to facilitate methylation by another more active subunit as we have observed with PRMT1 (8, 34), or a combination of both.

In this study, we sought to better understand how PRMT2 interacts with histones and other PRMT enzymes to determine whether any alternative mechanisms of PRMT2-mediated histone methylation exist. We used thermal shift assays to evaluate whether PRMT2 can bind to histone peptides and to assess heteromeric PRMT2 protein–protein interactions. These thermodynamic findings led us to identify *in vitro* conditions where PRMT1/2 interact (34, 35), which we confirmed by using fluorescently tagged PRMT1/2 enzymes and native PAGE analysis. We complemented these techniques by demonstrating PRMT2’s influence on the catalytic activity of PRMT1 towards calf thymus histones, independent histones, histone octamers, and mononucleosomes. Our findings demonstrate that PRMT2 enhances PRMT1 catalytic activity towards histone substrates.

## Results

### PRMT2 thermal stability characterization

Due to the low activity of recombinant human PRMT2 (8, 10), we sought a nonenzymatic method for characterizing ligand binding using purified proteins (Fig. S2). We chose to analyze PRMT2 interactions using differential scanning fluorimetry (DSF), a method that quantifies protein-ligand binding events using a hydrophobic reporter dye, SYPRO Orange (Fig. S3 and Tables S1–S4) (36, 37, 38). In our experiments, we incubated PRMT2 with SYPRO Orange and measured the dye’s fluorescent output as a function of temperature (Fig. S3*A*). Proteins unfold from thermal stress and reveal hydrophobic patches to which SYPRO Orange binds in DSF, resulting in augmented fluorescence; the melting temperature (T_m_) is the steepest point during the unfolding transition, and it is more clearly viewed in a first-derivative plot of a melt curve (36, 37, 38). PRMT2 produced a biphasic melting curve with lower- and higher-temperature melting transitions (Fig. S3*B*), T_m1_ and T_m2_, suggesting two discrete populations that could be constituted by melts of different conformations or oligomeric states. The fact that PRMT2 exhibited a concentration-dependent positive change in T_m_ values (Figs. S3*C* and S4*A*) points towards accompanying changes to the oligomeric state. The improved PRMT2 stabilization at higher enzyme concentrations can be partially attributed to glycerol in the PRMT2 storage buffer, which caused some PRMT2 thermal stabilization where T_m2_ became the major peak (Figs. S3*D* and S4*B*).

### PRMT2 ligand binding characterization

**Figure 1:**
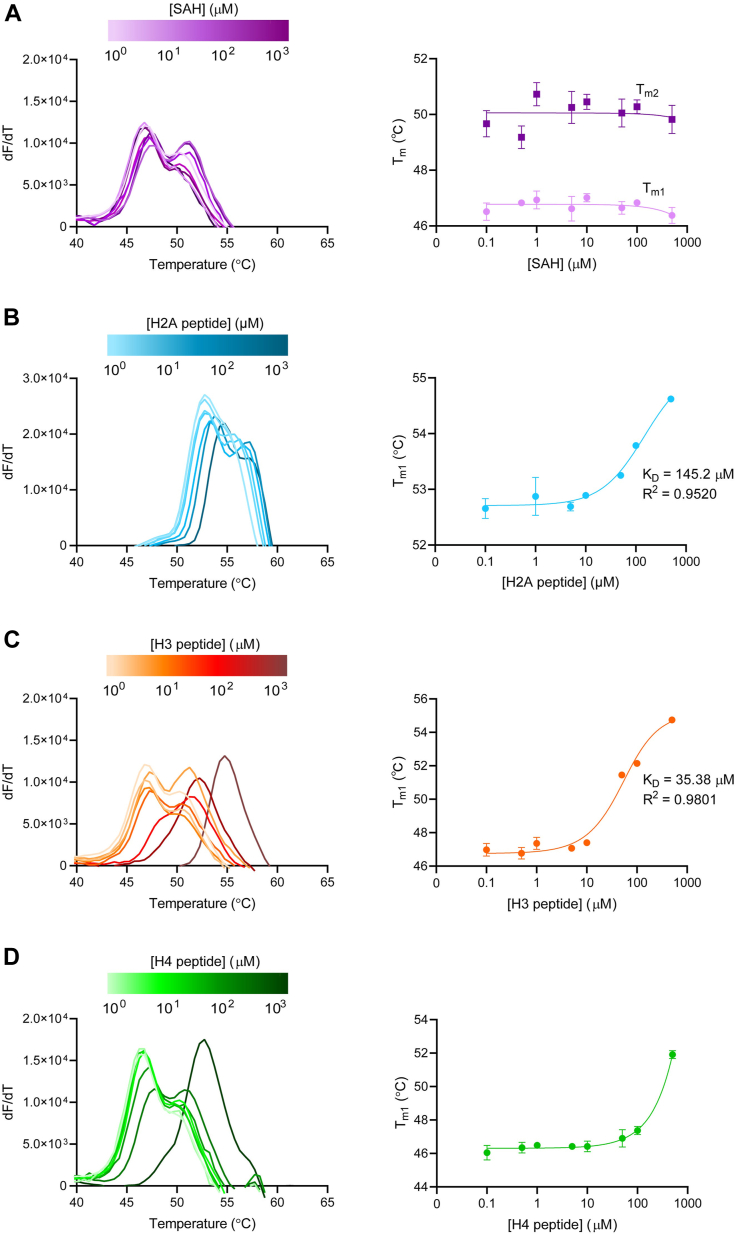
**Effects of ligand binding on PRMT2 thermal stability.** PRMT2 was incubated with increasing amounts of (*A*) SAH, (*B*) H2A peptide, (*C*) H3 peptide, or (*D*) H4 peptide for DSF measurements. First-derivative melting curves (in relative florescence units per second (RFU/s)) are shown on the *left*, while notable T_m_ trends for each of the ligands with PRMT2 are shown on the *right*. DSF-derived K_D_ was determined for H3 peptide binding to PRMT2. Data presented are averages of three replicates; error bars show SDs. Raw fluorescence spectra are available in Fig. S5. DSF, differential scanning fluorimetry.

Our binding efforts were focused on ligands described in previous studies indicating they may bind directly to PRMT2: SAH (8), histone H2A peptide (39), histone H3 peptide (11), and H4 peptide (8). We monitored both PRMT2 melting populations (*i.e.*, T_m1_ and T_m2_) over a broad range of ligand concentrations (Figs. 1, S5, and Tables S3 and S4). We noted some batch-to-batch variability in PRMT2 response due to different glycerol percentages per stock protein concentration, but this issue did not appear to affect relative thermal shifts. SAH failed to elicit thermally stabilizing responses towards either of the PRMT2 T_m_ values (Fig. 1*A*). All histone peptides caused a dose-dependent condensation of the two melting populations into a single T_m_ with concomitant thermal stabilization (Fig. 1, *B*–*D*). The histone peptides exhibited melting characteristics with Sypro Orange independent of PRMT2 (all peptides yielded 10-fold lower fluorescence and indistinct T_m_ values without PRMT2). Hence, we conclude that thermal stabilization observed was due to the interaction between PRMT2 and histone peptide (Fig. S5*E*). Compared to the apo state, H2A, H3, and H4 peptides at 500 μM each elicited ΔT_m_ of +1.97 °C, +7.76 °C, and +5.87 °C for ligand-bound PRMT2, respectively. By analyzing T_m1_ of PRMT2 in the presence of histone H2A and H3 peptides, DSF-derived K_D_ values of 145.2 and 35.38 μM were determined (Fig. 1, *B* and *C*). A K_D_ for histone H4 peptide binding to PRMT2 could not be generated due to poor model fitting (Fig. 1*D*). We additionally tested whether PRMT2 could bind both SAH and H3 peptide together by DSF (Fig. S6 and Tables S5 and S6). Compared to PRMT2 with H3 peptide, no change in T_m_ was seen, but the first-derivative peak height increased, indicating a steeper melt transition for the complex.

Given the lack of responsive from PRMT2 to SAH *via* DSF, we sought alternative approaches to evaluate whether PRMT2’s interaction with SAH is important for the histone peptide thermal stabilization. We made a PRMT2 SAM-binding mutation, H112Q, that has previously been shown to inhibit PRMT2 catalytic activity in cells (12). Like native PRMT2, PRMT2H112Q also displayed clearly biphasic melting properties, but the second melting transition was too broad to accurately define a melting temperature (Fig. S7*A*). The PRMT2H112Q T_m1_ was 2.4 °C lower than PRMT2 T_m1_ and the melt was overall much lower in raw fluorescence with gradual changes in fluorescence than the steeper transitions observed for WT PRMT2 (Fig. S7*B*). When PRMT2H112Q was incubated with either SAH and/or H3 peptide (saturating amounts), there was no statistically significant change in melting transition observed compared to the apo-state (Tables S7 and S8). No K_D_ measurement was performed for PRMT2H112Q with H3 peptide due to the lack of responsiveness at saturating H3 peptide.

### SH3 domain effects on PRMT2 thermal stabilization

To determine whether the PRMT2 SH3 domain played a role in binding to the histone H3 peptide, we performed DSF on the PRMT2 construct cloned without the SH3 domain (PRMT2ΔSH3). Unlike the full-length construct, PRMT2ΔSH3 produced a monophasic melt curve with very gradual changes in fluorescence (Fig. S8*A* and Tables S9–S11). This broad fluorescence change made differentiation of data difficult to interpret; therefore, data were normalized and fit to a Boltzmann sigmoidal nonlinear regression equation in which the T_m_ is the temperature value at 50% of maximum fluorescence (Fig. S8, *B* and *C*). The T_m_ obtained for PRMT2ΔSH3 was 39.5 °C, 6.98 °C lower than full-length PRMT2 T_m1_ at 46.5 °C (Fig. S8*D* and Tables S9–S11). When incubated with histone H3 peptide, PRMT2ΔSH3 was stabilized by 2.64 °C with 500 μM H3 peptide (no significant shift below this concentration) compared to 7.76 °C stabilization for full-length PRMT2 with 500 μM H3 peptide. Like full-length PRMT2, SAH negligibly impacted the melt curve of PRMT2ΔSH3 either in the presence or absence of H3 peptide. A K_D_ greater than 1400 μM was estimated from T_m_ values for the H3 peptide binding to PRMT2ΔSH3 (Fig. S8*E* and Tables S9–S11).

To further validate that the PRMT2 SH3 domain can bind to H3 peptide, we attempted DSF on the PRMT2 SH3 domain only (without the PRMT scaffold, amino acids 1–103). Unfortunately, there were no fluorescence differences between the SYPRO Orange dye alone and dye with SH3 domain (measured at multiple concentrations ranging from 100 nM to 100 μM data not shown), indicating that DSF could not be used to measure peptide binding to the SH3 domain. We attempted isothermal titration calorimetry on the PRMT2 SH3 domain with both H3 and H4 peptides and observed negligible changes in the heat of binding (data not shown).

### PRMT1/2 complex response to ligands

Our group previously demonstrated that PRMT2 can bind to PRMT1 *in vitro* using co-immunoprecipitation and in cells using biomolecular fluorescence complementation (BiFC), as well as increase PRMT1 activity *in vitro* towards histone H4R3, suggesting that these two enzymes can interact (34). We sought to investigate whether the PRMT1/2 complex can be thermodynamically characterized by DSF. We conducted control experiments on PRMT1 in the absence of PRMT2 to evaluate how it interacts with the H3 peptide with or without SAH. PRMT1 showed a small thermal stabilization in the presence of 500 μM H3 peptide and a much greater increase in stabilization when H3 peptide and SAH were present at 500 μM each (Fig. S9). We determined a DSF-derived K_D_ for H3 peptide binding to PRMT1 of 105.0 μM in the presence of 500 μM SAH (Fig. S9 and Tables S12–S14).

**Figure 2:**
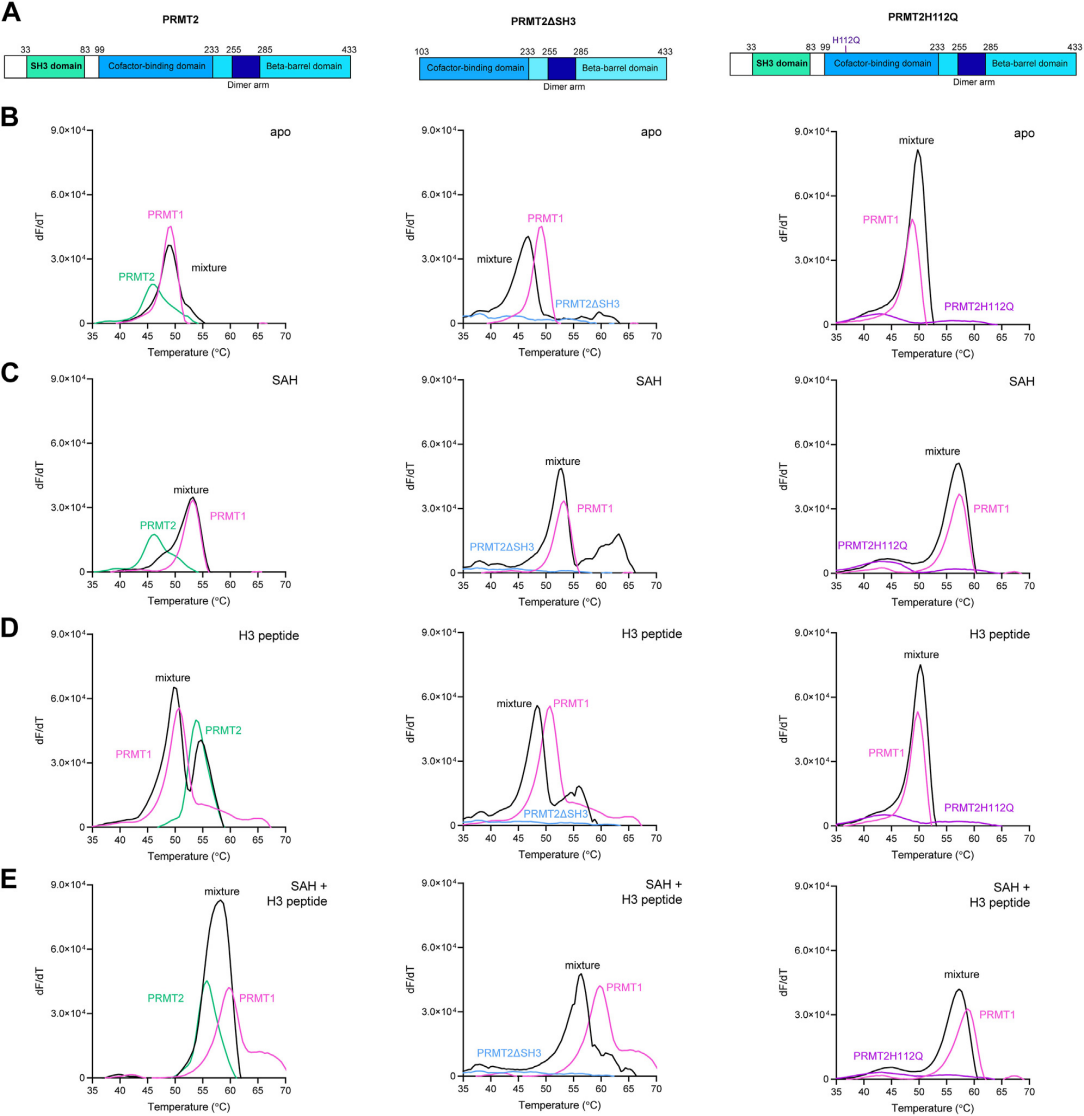
**PRMT1/2 response to ligands by DSF.***A*, modular schematics depicting the three PRMT2 variants evaluated in complex with PRMT1. The PRMT2ΔSH3 protein contains a 102-amino acid deletion of the PRMT2 N terminus. PRMT2H112Q has a single point mutation at position 112 (marked with a *purple* line). First-derivative melting curves (in relative florescence units per second (RFU/s)) of PRMT1 and PRMT2 (*left*), PRMT2ΔSH3 (*middle*), or PRMT2H112Q (*right*) were evaluated by DSF (*B*) in the absence of ligands, (*C*) presence of 500 μM SAH, (*D*) presence of 500 μM H3 peptide, or (*E*) presence of both SAH and H3 peptide at 500 μM each (n = 3). Raw fluorescence spectra are available in Figs. S10–S12. DSF, differential scanning fluorimetry; SH3, Src-homology 3.

We used DSF on PRMT1 with PRMT2 (PRMT1/2), PRMT2ΔSH3 (PRMT1/2Δ), and PRMT2H112Q (PRMT1/2H112Q) to probe for conditions that change protein thermal stability indicative of protein–protein interactions (Figs. 2, S10–S12, and Tables S15–S20) (40, 41). In the absence of SAM and H3 peptide, the mixed thermal stability of PRMT1/2 and PRMT1 alone showed no differences, and the PRMT1/2Δ mixture yielded a biphasic melt with the major peak between the melting temperatures of PRMT1 and PRMT2ΔSH3 (Fig. 2*B*). In contrast, the PRMT1/2H112Q mixture yielded a biphasic melt with a small but evident shoulder (T_m1_) that was 1.61 °C lower than PRMT2H112Q and the major peak (T_m2_) that was 0.9 °C higher than PRMT1 T_m1_ (Fig. 2*B*). In the presence of saturating SAH concentration, we saw no difference between the T_m_ of the PRMT1/2 population and PRMT1 (Fig. 2*C* and Table S16). The thermal melt of the PRMT1/2Δ mixed population with SAH had two distinct melting temperatures, where T_m1_ was close to PRMT1 with SAH, but T_m2_ was greater than the T_m_ values of each individual PRMT melt with SAH (Fig. 2*C* and Table S8). The PRMT1/2H112Q mixture yielded biphasic curves in the presence of saturating amounts of either SAH or H3 peptide; neither peak showed significant differences in thermal stability compared to the individual PRMT melts (Fig. 2, *C* and *D* and Table S20). The addition of a saturating H3 peptide concentration to PRMT1/2 or PRMT1/2Δ yielded two distinct peaks in the first-derivative plot that mimicked peaks seen for each enzyme alone with H3 peptide (Fig. 2*D* and Table S16). In Figure 2*E*, both H3 peptide and SAH incubated with PRMT1/2 resulted in a single sharp peak representative of a steep monophasic melt transition that appeared to combine T_m_ values from individual PRMTs. In the absence of the SH3 domain, however, the peak height of PRMT1/2Δ with both ligands was not as sharp and with a lower T_m_ than what is seen for PRMT1/2, suggesting that the SH3 domain adds to complex stability in response to H3 peptide and SAH (Fig. 2*E* and Table S18). The PRMT1/2H112Q mixture in the presence of saturating H3 peptide and SAH yet again yielded a biphasic melting transition, but T_m1_ was 1.68 °C more stable than PRMT2H112Q, and T_m2_ was 1.54 °C less stable than PRMT1 (Fig. 2*E* and Table S20). We additionally evaluated how varying the concentration of PRMT1 (1–10 μM) while holding PRMT2 constant at 10 μM and vice versa impacts the changes in melting transitions in the different conditions listed above (Fig. S13 and Table S21). We found that PRMT1 and PRMT2 influenced each other’s thermal stabilities primarily in the presence of both SAH and H3 peptide.

**Figure 3:**
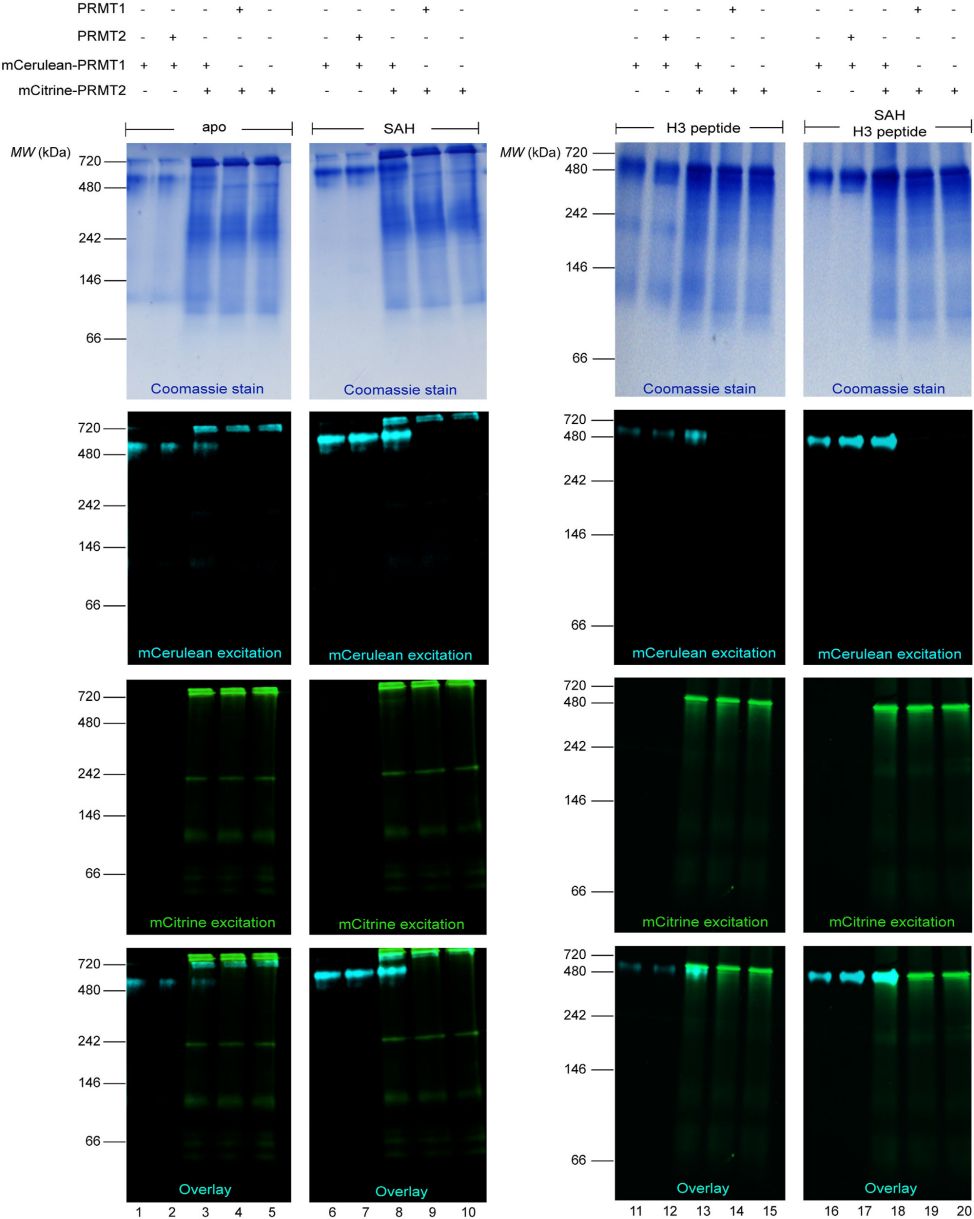
**Native PAGE gels of fluorescent PRMT1/2 heteromeric complexes.** PRMTs each at 1 μM were incubated for 1 h at 37 °C in the presence or absence of SAH (10 μM) and/or histone H3 peptide (200 μM). The proteins were separated by electrophoresis on a 4 to 20% native PAGE gel and imaged to detect fluorescent proteins at their specific excitation wavelengths. The overlay is a composite of fluorescent images.

The evidence that PRMT1 and PRMT2 can influence one another’s thermal stability in a cofactor- and domain-dependent manner prompted us to determine whether a PRMT1/2 interaction could be observed with mCerulean (mCer) and mCitrine (mCit) fluorescent fusions of PRMT1 and PRMT2 using native PAGE (Fig. 3). We used substrate concentrations above K_I_ and K_M_ values for PRMT1 towards SAH and H3 peptide, respectively (10). In the absence of SAH or H3 peptide, both mCer-PRMT1 and mCit-PRMT2 displayed multiple banding patterns consistent with monomeric, dimeric, and higher order oligomers with no overlap between mCer or mCit signals when the two enzymes were mixed. The addition of SAH caused mCer-PRMT1 bands to condense into one single band at ∼450 kDa, producing an increase in mCer signal compared to the apo state, whereas no mCit-PRMT2 banding pattern change was apparent upon SAH addition. The H3 peptide consolidated the higher fluorescent bands for mCit-PRMT2 to a uniform species around ∼600 kDa while also reducing the signal for the lower molecular weight species regardless of PRMT1 presence. The opposite is seen for mCer-PRMT1, where the H3 peptide caused a reduction in the highest band by Coomassie stain and an emergence of more signal at ∼70 and 150 kDa. When the fluorescent populations were mixed with H3 peptide, the emergence of a single band containing mCer and mCit emission was observed in the overlay (Fig. 3, lane 13). We saw a more pronounced, consolidated band bearing both mCer-PRMT1 and mCit-PRMT2 in the presence of both SAH and H3 peptide (Fig. 3, lane 18). These results mimic the same DSF responsiveness to ligands for PRMT1 and PRMT2 individually and in combination (Fig. 2).

### PRMT2 impact on PRMT1 substrate specificity

**Figure 4:**
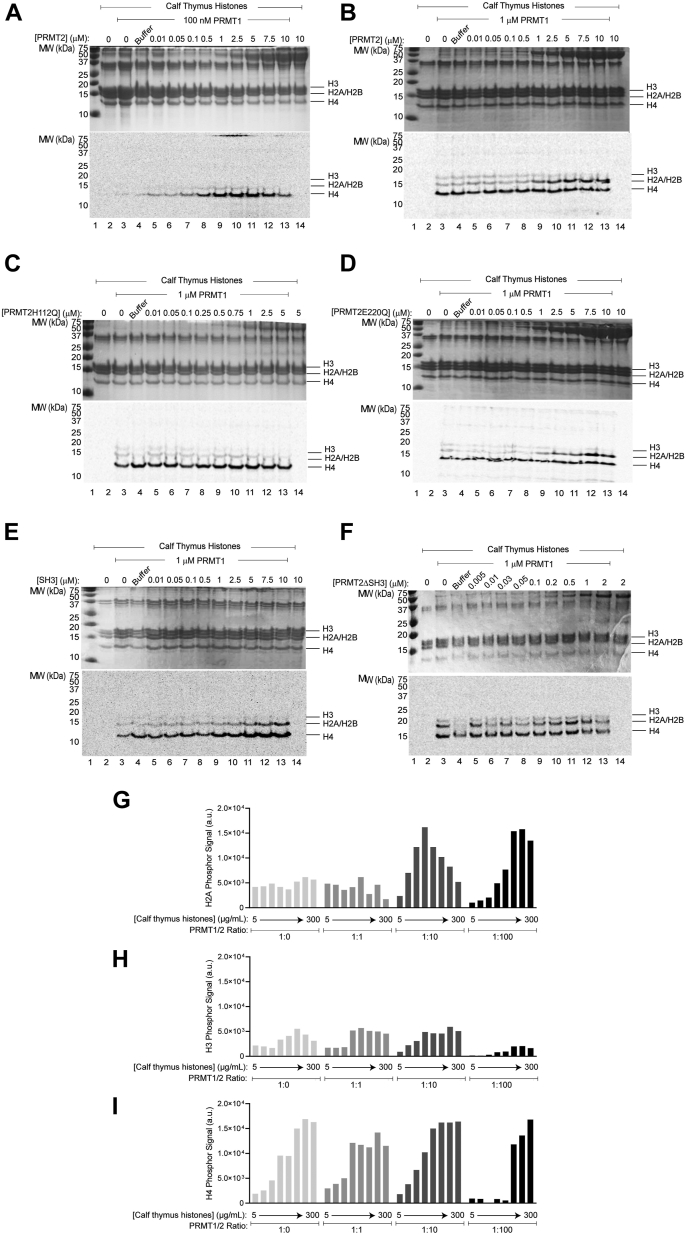
**PRMT1/2 methylation of calf thymus histones.***A*, PRMT1 (100 nM) or (*B*) PRMT1 (1 μM) methylation reactions using 10 μM ^14^C-SAM and 10 μg calf thymus histones with increasing amounts of PRMT2. Panels C-F are the same conditions as described in panel B with the following PRMT2 variants: (*C*) PRMT2H112Q, (*D*) PRMT2E220Q, (*E*) PRMT2 SH3 domain, (*F*) PRMT2ΔSH3. Reactions were separated on a 16.5% Tricine SDS-PAGE and visualized by Coomassie staining (*top*), followed by phosphor imaging analysis after a 24-h exposure (*bottom*). Phosphor signal from PRMT1 (100 nM) methylation towards histone (*G*) H2A, (H) H3, or (I) H4 as a function of increasing calf thymus histone amount in the presence of increasing amounts of PRMT2 (0, 100, 1000, 10,000 nM). Raw data for panels *G*–*I* are available in Fig. S16 and Table S22. SH3, Src-homology 3.

To assess whether the interaction between PRMT1 and PRMT2 impacts PRMT1 enzymatic activity, we evaluated the methylation activity of PRMT1/2 (at varying molar ratios) towards calf thymus histones using ^14^C-SAM (Figs. 4, S14–S16, and Table S22). Calf thymus histones were used as an initial screening substrate given its low cost, ease of sample preparation, and presence of multiple arginine-rich proteins. To visualize and distinguish methylation of the individual histones, we separated reactions by gel electrophoresis on 16.5% Tricine-SDS-PAGE gels that were subsequently stained with Coomassie blue to visualize proteins, and the dried gels were exposed to phosphor screens to quantify ^14^C-methylated substrate.

Our first experiment was similar to our previous study, where we incubated 100 nM PRMT1 with increasing amounts of PRMT2 (from 10-10000 nM) and monitored histone H4 methylation, but here we used calf thymus histones (Fig. 4*A*). In the absence of PRMT2, PRMT1 methylation activity towards histone H4 was barely detectable, and PRMT2 activity at 10 μM was not detectable. With increasing PRMT2 concentrations in the presence of PRMT1, the H4 methylation signal concomitantly increased up to 5 μM PRMT2; higher PRMT2 concentrations resulted in a decreased H4 methylation (Fig. 4*A*, lanes 12 and 13). Detectable levels of histone H2A methylation can also be seen in the phosphor image. We performed a control experiment with a titration of PRMT2 storage buffer and observed decreased H4 methylation at high glycerol concentrations, indicating increased H2A and H4 methylation is PRMT2-specific (Fig. S14). We repeated the same experiment with 10-fold more PRMT1 (1 μM) while keeping all other variables constant (Fig. 4*B*). At this concentration, PRMT1 alone predominantly methylated histone H4 but also methylated histones H3 and H2A to a lesser extent (Fig. 4*B*, lane 3). With increasing PRMT2 against 1 μM PRMT1, histone H4 methylation remained the same while histone H2A methylation dramatically increased and histone H3 methylation decreased (Fig. 4*B*, lanes 4–13). These results demonstrate that PRMT2 can influence PRMT1 activity in a concentration-dependent manner. For this reason, we refer to this experiment as the “influencer assay.” We performed control reactions with a catalytically inactive PRMT1E153Q and observed no histone methylation, even in the presence of PRMT2, confirming that histone methylation is dependent on PRMT1 activity (Fig. S15).

To further probe the mechanism of how PRMT2 influences PRMT1 activity, we performed the influencer assay on PRMT1 with PRMT2 mutants. The SAM-binding mutant PRMT2H112Q did not show any effect on PRMT1 methylation of calf thymus histones (Fig. 4*C*), demonstrating that the ability of PRMT2 to bind SAM is critical for how it influences PRMT1 activity. PRMT2E220Q, previously demonstrated to inhibit PRMT2 methylation activity (34), showed the exact same pattern of enhanced H2A methylation as did WT PRMT2 (Fig. 4*D*). Thus, PRMT2 catalytic activity is not required for it to influence PRMT1 methylation of histones H4 and H2A. The two truncations of PRMT2 we tested were the PRMT2 SH3 domain alone and PRMT2 without its SH3 domain (PRMT2ΔSH3). When we performed the influencer assay using the PRMT2 SH3 domain against PRMT1, we saw a dose-dependent increase in the methylation of both histones H2A and H4 (Fig. 4*E*), indicating that the PRMT2 SH3 domain alone can augment PRMT1 activity towards selective histone substrates. Without the SH3 domain, PRMT2ΔSH3 failed to improve PRMT1 activity above the activity of PRMT1 alone (Fig. 4*F*).

We sought to evaluate how different ratios of PRMT1/2 (1:0, 1:1, 1:10, 1:100) are able to methylate each of the calf thymus histones as a function of increasing substrate concentrations (Fig. 4, G–I). This was done by performing densitometry on the phosphor images from the gels (Fig. S16 and Table S22). At highest PRMT2 concentrations (1 and 10 μM) and lowest calf thymus histone concentrations, PRMT1 methylated PRMT2 to a similar extent as histone H4 from calf thymus histone, but PRMT2 methylation was inhibited at higher histone concentrations (Fig. S16). Only after a 10-fold PRMT2 excess of PRMT1 did we see noticeable changes in the histone methylation pattern, specifically a three-fold increase in the methylation signal of histone H2A at 50 μg/ml calf thymus histones (Fig. 4*G*). At a 100-fold PRMT2 excess, the increase in histone H2A methylation signal occurred after 100 μg/ml (Fig. 4*G*), histone H3 methylation signal decreased over all substrate concentrations compared to other PRMT1/2 ratios (Fig. 4*H*), and the highest substrate concentrations were required to yield histone H4 methylation signals achieved with lower PRMT1/2 ratios (Fig. 4*I*). Due to the heterogenous nature of calf thymus histones purified from eukaryotic cells and bearing posttranslational modifications, we were unable to use this data to derive any quantitative enzymatic properties from these gels.

**Figure 5:**
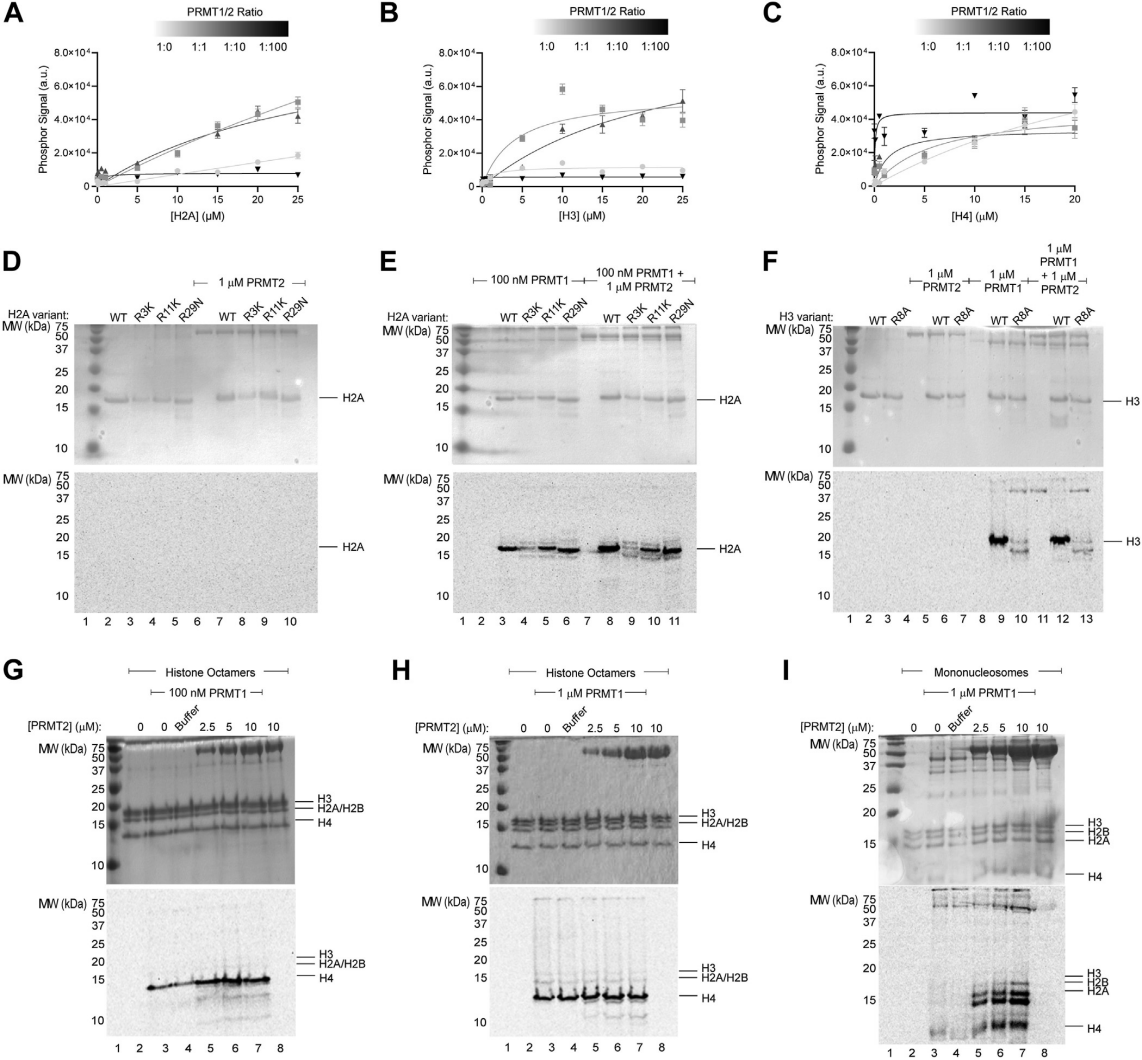
**PRMT1/2 methylation of histones in different macromolecular contexts.** PRMT1 (100 nM) methylation reactions (2 h) with 0, 100, 1000, or 10,000 nM PRMT2 towards recombinant histones (*A*) H2A, (*B*) H3, and (*C*) H4 using 10 μM ^14^C-SAM and signal quantification by FBAPS. *D*–*E*, PRMT1 (100 nM) and/or PRMT2 (1 μM) methylation reactions (2 h) towards recombinant histone H2A mutants using 10 μM ^14^C-SAM. *F*, PRMT1 (1 μM) and/or PRMT2 (1 μM) methylation reactions (2 h) towards recombinant histone H3 mutants using 10 μM ^14^C-SAM. *G*, PRMT1 (100 nM) or (*H*) PRMT1 (1 μM) methylation reactions (16 h) using 10 μM ^14^C-SAM and 2.5 μM recombinant H3.3 histone octamer with increasing amounts of PRMT2 (12-h exposure for phosphor image). *I*, PRMT1 (1 μM) methylation reactions (16 h) using 10 μM ^14^C-SAM and 1 μM recombinant H3.3 mononucleosomes with increasing amounts of PRMT2 (1-week exposure for phosphor image). Reactions were separated on a 16.5% Tricine SDS-PAGE and visualized by Coomassie staining (*top*), followed by phosphor imaging analysis (*bottom*). Raw data for panels *A*–*C* and densitometry for panel *E* are available in Tables S23–S26, respectively. FBAPS, filter binding and phosphor screening.

Our next set of experiments were designed to evaluate how PRMT2 can influence PRMT1 activity towards purified and unmodified histone proteins in different macromolecular contexts. We expressed and purified histones H2A, H3, and H4 for *in vitro* PRMT1/2 methylation analysis. In these experiments, we titrated the protein substrate against different molar ratios of PRMT1:PRMT2 (1:0, 1:1, 1:10, 1:100) and measured methyl transfer by the filter binding and phosphor screening (42) for individual histones (Figs. S17–S19). Densitometry data for reactions are presented after subtracting phosphor signal from the histone-only control. We observed no detectable signal for reactions with PRMT2 alone (Fig. S20). Without PRMT2, PRMT1 methylation activity towards histone H2A was best fit to a simple linear regression, indicating the K_M (App)_ could be beyond the concentrations tested (Fig. 5*A*). In the presence of 1:1 and 1:10 PRMT1/2 ratios, H2A methylation data fit to a nonlinear regression model (Michaelis–Menten) where the K_M (app)_ decreased to 128 and 44.7 μM at 1:1 and 1:10 PRMT1/2 ratios, respectively (Table 1). The total methylation signal for PRMT1 towards H2A increased by several fold at 1:1 and 1:10 PRMT1/2 ratios compared to PRMT1 alone (Figs. 5*A* and S17). Based on the Michaelis–Menten fit, there is a dose-dependent decrease in *k*_cat (App)_ for the PRMT1 methylation of H2A as the PRMT1/2 ratio increased above 1:1 (Table 1). A similar trend in *k*_cat (App)_ increase was also observed when PRMT2 was added to PRMT1 methylation reactions against histone H3, except for the 1:100 ratio where methylation appeared inhibited by PRMT2 (Fig. 5*B*, Table 1 and Fig. S18). The increase in *k*_cat_ for PRMT1 methylation of H3 with increasing PRMT2 is also accompanied by a dose-dependent increase in K_M_ for 1:1 and 1:10 ratios (Fig. 5*B* and Table 1). Accordingly, the apparent specificity constant *k*_cat_/K_M__(App)_ remained relativity constant for histone H2A with PRMT1/2 at ratios of 1:1 to 1:10 but increased nearly 5-fold at the 1:100 ratio. In contrast, *k*_cat_/K_M__(App)_ for histone H3 was similar with PRMT1 only and the 1:1 presence of PRMT2 but decreased at least 2.7-fold at a 1:10 PRMT1/2 ratio. PRMT1 methylation of histone H4 in the absence of PRMT2 had the highest *k*_cat (App)_ of all the substrates tested, as expected based on the gel radiography and previous studies (Fig. 5*C* and Table 1) (10, 34, 43). As PRMT2 was introduced to the PRMT1 methylation reactions with histone H4, there was an increase in phosphor signal (Figs. 5*C* and S19). With increasing PRMT2, there was a trend of decreasing K_M (App)_ values while the *k*_cat (App)_ remains relatively consistent, but the data fit poorly to the Michaelis–Menten model (Fig. 5*C* and Table 1). We attempted to measure the activity of PRMT1/2 towards lower histone H4 concentrations to determine whether PRMT2 can decrease the PRMT1 K_M (App)_ for histone H4, but unfortunately PRMT1 methylation of PRMT2 dominated the signal (like that observed in Fig. S16). Given the data presented for histone H4, the *k*_cat_/K_M__(App)_ for histone H4 increased roughly by 2-, 6-fold at 1:1 and 1:10 PRMT1/2 ratios above PRMT1 alone, but a different method is required to evaluate nanomolar histone H4 methylation reactions to obtain accurate fits (Table 1).

**Table 1:** Tabulated apparent kinetic parameters for PRMT1/2 methylation of histones H2A, H3, and H4

Substrate	PRMT1/2 ratio											
	PRMT1	PRMT1/2 (1:1)	PRMT1/2 (1:10)	PRMT1/2 (1:100)	PRMT1	PRMT1/2 (1:1)	PRMT1/2 (1:10)	PRMT1/2 (1:100)	PRMT1	PRMT1/2 (1:1)	PRMT1/2 (1:10)	PRMT1/2 (1:100)
	H2A				H3				H4			
*k*_cat (App)_ (× 10^−6^ s^−1^)	n/a	26.6 ± 7.41	10.7 ± 1.65	0.480 ± 0.03	1.02 ± 0.04	5.44 ± 0.36	10.9 ± 0.38	n/a	267 ± 170	79.8 ± 21.8	76.2 ± 3.99	n/a
K_M (App)_ (× 10^−6^ M)	n/a	128 ± 36.8	44.7 ± 6.53	0.456 ± 0.23	0.992 ± 0.10	4.02 ± 0.35	28.2 ± 11.5	n/a	38.8 ± 13.9	6.22 ± 3.42	1.66 ± 0.98	n/a
*k*_cat_/K_M__(App)_ (M^−1^s^−1^)	n/a	0.208 ± 0.083	0.239 ± 0.051	1.05 ± 0.54	1.03 ± 0.11	1.35 ± 0.15	0.387 ± 0.16	n/a	6.87 ± 5.02	12.8 ± 7.85	33.7 ± 20.4	n/a
R^2^	n/a	0.962	0.805	0.519	0.802	0.848	0.955	n/a	0.961	0.718	0.380	n/a

Data from Figure 5, *A*–*C* were used for calculations. The data for the PRMT1 (PRMT1/2 ratio 1:0) methylation of H2A, PRMT1/2 (1:100) methylation of H3, and PRMT1/2 (1:100) methylation of H4 were fit by simple linear regression analysis yielding the following lines of best fit: H2A: y = 0.01461x + 7.733 (R^2^ = 0.941); H3: y = 96.59x + 4273 (R^2^ = 0.501); H4 y = 1055x + 32,391 (R^2^ = 0.546), respectively. Adjusted correlation coefficients (R^2^) corresponding to nonlinear regressions fit to (Equation 3) for H2A, H3, and H4 data are indicated.

Our next aim was to determine whether PRMT2 changes the site of methylation for PRMT1 on histones. We previously demonstrated that H4R3 is the primary site of augmented arginine methylation by PRMT1/2 showing that the H4R3K peptide was unable to be methylated (34). Using site-directed mutagenesis, we made the following histone N-terminal tail mutations: H3R8A, H2AR3K, H2AR11K, and H2AR29N. These purified proteins were assayed to determine the major methylation site for PRMT1/2. We evaluated the activity of PRMT1, PRMT2, and PRMT1/2 against WT and mutant versions of these histones by gel radiography to visualize changes in activity (Fig. 5, *D* and *E*). Compared to WT histone H2A, H2AR3K resulted in the greatest reduction in activity of the three mutants by either PRMT1 or PRMT1/2 (Fig. 5*E* and Table S26). Although H2AR3K showed reduced methylation, some residual signal was detectable, indicating that there could be low-level methylation at other sites. H2AR11K also decreased methylation compared to WT H2A, although to a lesser extent than H2AR3K (Fig. 5*E* and Table S26). When we screened the histone H3R8A mutation against PRMT1 and PRMT1/2, there was a ten-fold reduction in methylation compared to WT histone H3, indicating R8 is the primary arginine methylated by PRMT1 and PRMT1/2 (Fig. 5*F*).

In order to present histone substrates in a more biologically relevant context, we tested the PRMT2 influencer assay on different histone complexes. We performed exhaustive methylation reactions with 100 nM or 1 μM PRMT1 at different PRMT2 concentrations against recombinant histone H3.3 octamers (Fig. 5, *G* and *H*). At both PRMT1 concentrations, PRMT2 appeared to elicit an increase in PRMT1 activity towards histone H4 and to a lesser degree, other lower molecular weight contaminants. In contrast, the exhaustive methylation of unmodified recombinant nucleosomes with 1 μM PRMT1 revealed a robust PRMT2-dependent increase in the methylation of histone H2A and H4, as well as methylation of an unknown partial degradation product that migrated just below H2A (Fig. 5*I*). At 10 μM PRMT2 with 1 μM PRMT1, an increased methylation of histone H2B was also detected. These results indicate that PRMT2 influences the amount of PRMT1 methylation activity and histone substrate specificity within a recombinant nucleosome substrate context.

**Figure 6:**
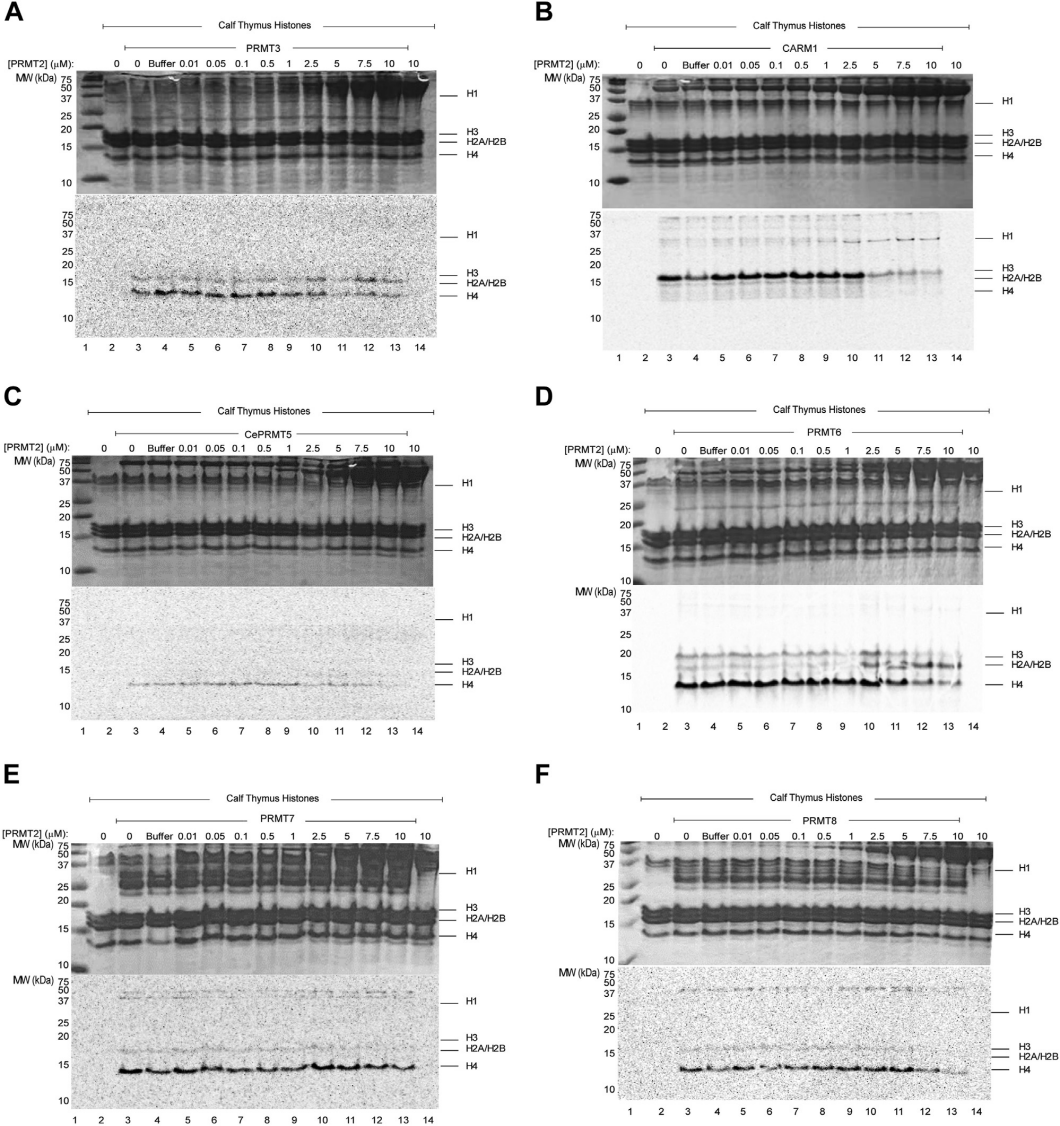
**PRMT2 influence on PRMT methylation of histones.***A*, crude PRMT3 (48-h exposure), (*B*) CARM1 (48-h exposure), (*C*) *Caenorhabditis elegans* PRMT5 (cePRMT5) (2-weeks exposure), (*D*) PRMT6 (24-h exposure), (*E*) crude PRMT7 (48-h exposure), and (*F*) PRMT8 (24-h exposure) (1 μg each) methylation reactions using 10 μM ^14^C-SAM and 10 μg calf thymus histones with increasing amounts of PRMT2. *E*, reactions were separated on a 16.5% Tricine SDS-PAGE and visualized by Coomassie staining (*top*), followed by phosphor imaging analysis (*bottom*). Exposure time for each phosphor image indicated next to corresponding enzyme used. CARM, coactivator-associated arginine methyltransferase; PRMT, protein arginine N-methyltransferase.

To determine whether the alteration in substrate specificity induced by PRMT2 towards PRMT1 was a unique phenomenon for this pairing, we repeated the same PRMT2 titration against PRMT3-8 with calf thymus histones as the substrate (Fig. 6 and Table 2). Among all combinations, high concentrations of PRMT2 inhibited histone H3 and H4 methylation (except for PRMT1). When PRMT2 was titrated against CARM1, we observed modest increases in the methylation of a band at ∼32 kDa consistent with the size of histone H1 (Fig. 6*B*). Reactions with PRMT6 showed a PRMT2 dose-dependent increase in histone H2A methylation (Fig. 6*D*). These results suggest that PRMT2 can influence other PRMTs and cause changes to histone methylation.

**Table 2:** PRMT methylation activity towards calf thymus histone**s** ± **PRMT2**

Enzyme	No PRMT2				PRMT2 (10 μM)			
	H1	H2A/H2B	H3	H4	H1	H2A/H2B	H3	H4
PRMT1	−	+	+	+++	−	+++	−	+++
PRMT3	−	+	−	++	−	+	−	+
PRMT4	−	+	+++	−	+	−	+	−
cePRMT5	−	−	−	++	−	−	−	−
PRMT6	−	+	++	+++	−	+++	−	++
PRMT7	−	−	+	++	−	−	−	++
PRMT8	−	+	−	+++	−	−	−	+

Recombinant PRMT enzymes were purified to varying extents and precomplexed with PRMT2 for 1 h at 37 °C (∗except cePRMT5, which was incubated at 25 °C) prior to a 2 h incubation with 10 μg calf thymus histone and 10 μM ^14^C-SAM at 37 °C. Proteins were separated on 16.5% tricine-SDS-PAGE gel, visualized by Coomassie stain, and methyl transfer was analyzed by phosphor imaging (exposure times available in Fig. 6). The reported methylation patterns are relative to the respective gel/enzyme.

## Discussion

### PRMT heteromeric complexation

The formation of PRMT heteromeric complexes is not a novel observation. The first investigations into interactions between PRMT2 and other PRMTs employed transient expression of GFP fusions of PRMT1-8 in HEK293 cells stably expressing GFP-PRMT2 (44). Immunoprecipitation (IP) of PRMT2 and Western blotting against GFP showed that PRMT2 was able to pull down GFP-PRMT8, which had previously been shown to be mediated between PRMT2’s SH3 domain and two proline-rich stretches in PRMT8’s N terminus (45), as well as faint bands consistent with GFP-PRMT1 and GFP-PRMT6. Our group identified permissible conditions for the formation of a PRMT1/6 complex *in vitro* using GFP mutants (mCer and mCit), which were used to quantitate PRMT hetero- and homo-oligomerization by FRET analysis (46). A recent study added CARM1 to the list of PRMT enzymes capable of forming heteromeric complexes with other PRMT enzymes (47). Using IP techniques, these authors demonstrated that PRMT2 and PRMT4 can pull down one another from MCF7 cells along with the BRD4 protein (47). It was further shown that CARM1 methylation of BRD4 was influenced by PRMT2, as upregulation of PRMT2 increased its methylation, and siRNA silencing of PRMT2 led to decreased methylation (47). In parallel with the discovery of the PRMT2/4 complex, another group identified PRMT5 and PRMT6 as functionally associating enzymes as oncogenic drivers in colorectal cancer (48). This growing body of evidence challenges the view of PRMT enzymes as solo writers of the methylarginine mark and instead recognizes that these enzymes can act in concert to modulate their methylation activities.

Our group investigated heteromeric PRMT1/2 interactions both *in vitro* and in HeLa cells using co-immunoprecipitation, BiFC, and enzymatic assays (34). PRMT1/2 co-immunoprecipitated one another *in vitro* and in HeLa cells. BiFC showed that PRMT1/2 colocalized in HeLa cells in an SH3 domain–dependent fashion (34). Our enzymatic assays demonstrated enhanced PRMT1 activity towards recombinant histone H4 *in vitro* in a PRMT2 concentration-dependent fashion and a global increase in MMA, aDMA, and symmetric dimethylarginine levels on total cellular protein with ectopic expression of PRMT1 and PRMT2 in HeLa cells (34). Our findings led us to propose that PRMT2 can form a heteromeric complex with PRMT1 and possibly other PRMTs.

In this study, we used DSF, native gel electrophoresis with fluorescently tagged enzymes, and activity assays to identify conditions where PRMT1/2 form favorable interactions. DSF has been previously used to study protein complex formation by comparing the thermal stability of a complexed state to that of a noncomplexed state (40, 41). Our group previously established conditions for measuring cofactor and peptide ligand binding to human PRMT1 *in vitro* using the DSF assay (37), and Cavarelli’s group performed ligand-binding studies on murine PRMT2 *via* DSF, revealing thermal stabilization of the protein at 1 mM concentrations of SAH, sinefungin, and the bisubstrate analog Cp1 relative to the apo protein (9).

### Human PRMT2 thermal stability characterization

Proteins displaying multiphasic melting properties in DSF may be indicative of impurities, independently-folded domains, or oligomeric populations with distinct thermal stabilities (37, 49, 50). In this study, we found that human PRMT2 provided a largely biphasic melt depending on protein concentrations. The more thermally stable population (T_m2_) became dominant in the presence of increased protein concentration, which we postulated was due to concentration-dependent PRMT2 homo-oligomerization common among PRMTs (46). The PRMT2 thermal stabilization observed with glycerol can be attributed to the kosmotropic effect of glycerol on colloidal protein stability (51). The utility of DSF is limited in that it does not allow for any assessment of the oligomerization state of each melting population. For this reason, we chose to complement DSF with other techniques like native PAGE gel electrophoresis to gain further insight into higher order PRMT structures and responsiveness to ligands.

PRMT2 melts varied considerably in response to different ligands. Unlike PRMT1 and murine PRMT2 (9), human PRMT2 did not display thermal stabilization in the presence of SAH. A close comparison of human and murine PRMT2 sequences (Fig. S21) reveal differences between residues in the cofactor-binding domain, the SH3 domain, and the dimerization arm that may account for any thermal stability differences in response to SAH. Another possibility may be attributed to differences in the DSF experiment itself (*e.g.*, different buffers and SAH concentrations used). A novel finding for this study was that histone peptides representative of the N-terminal tails of histone H2A, H3, and H4, all could elicit a thermally stabilizing response with PRMT2 (Fig. 1). The peptide-binding preference for PRMT2 was H3 > H2A > H4 *via* DSF. We have previously established an important mechanistic relationship between cofactor binding (SAM or SAH) and dimer formation (46), which did not appear to be the case for PRMT2 here. Due to the lack of responsiveness from PRMT2 to SAH in the presence or absence of histone H3 peptide, we cannot comment on the binding order of PRMT2 to its substrates using DSF. In lieu of a functional assay for measuring PRMT2 interaction with SAH, we evaluated how PRMT2H112Q, the PRMT2 SAM-binding mutation, could interact with ligands (12). The PRMT2H112Q completely inhibited the interaction between PRMT2 and histone peptides, indicating that the ability to bind to cofactor is required for peptide substrate binding.

### Noncanonical SH3 domain functions

The PRMT2 SH3 domain retains the same vital structural elements of a canonical SH3 domain, yet its amino acid sequence does not align well with sequences of other SH3 domains (52, 53). Several studies have demonstrated that the PRMT2 SH3 domain is capable of binding to canonical polyproline stretches, despite differences in sequence alignment (27, 28). Our DSF and methylation data suggest that the PRMT2 SH3 domain facilitates histone protein binding. Akin to the PRMT2 SH3 domain, the Mona Gads SH3 domain is capable of binding to both proline-rich as well as an R/KXXK motif where X is any amino acid (52, 54). We aligned SH3 domain structures of PRMT2 and Mona Gads complexed to its peptide ligand and speculate that basic residues within the R/KXXR/K motifs found in all four core histones can bind to the acidic regions of the PRMT2 SH3 domain (Fig. S22). Given our data for the PRMT2 and PRMT2ΔSH3, it appears that the SH3 domain contributes to the binding of PRMT2 to the histone peptides, but due to a lack of evidence demonstrating direct peptide binding, we cannot draw any conclusions from our results in this study. Alternative methods for measuring SH3 binding to these basic peptides will need to be investigated to identify a binding mechanism.

The role of the PRMT2 SH3 domain has remained elusive. Splice variants of PRMT2 bearing the SH3 domain and the cofactor-binding domain without an intact dimerization arm and beta-barrel domain (*i.e.*, C-terminal truncations) have been implicated in breast cancer (55, 56).These variants were shown to be capable of augmenting estrogen receptor alpha–mediated transactivation activity. Yet other studies have shown that the SH3 domain and much of the N-terminal portion of PRMT2 was not required for nuclear receptor binding and transcriptional activation (31, 57). Taken in the context of heteromeric PRMT2 complexes, it will be useful to see how different PRMT2 truncations may affect complex assemblies with nuclear receptors and other PRMTs.

### Requirements for PRMT1/2 complexation

Our previous work established that PRMT1 and PRMT2 can interact both *in vitro* and in cells (34); here we wanted to establish the molecular underpinnings responsible for this interaction. Using DSF, native gel electrophoresis, and enzymatic activity assays, we demonstrated evidence of a protein–protein interaction between PRMT1 and WT PRMT2. Our DSF data and native gel electrophoresis indicate that SAH and H3 peptide are required for PRMT1 and PRMT2 to interact. The most significant evidence for a PRMT1/2 protein–protein interaction is that PRMT2 influences PRMT1 catalytic activity towards specific histone proteins in a concentration-dependent manner. PRMT2 mutants revealed that PRMT2 SAM binding and SH3 domains play a role in mediating interactions between PRMT1, PRMT2, and histone protein substrates. Further mechanistic studies should be performed to map out binding interactions within this enzyme–substrate complex.

### PRMT2 impact on substrate specificity

Previously, our group demonstrated that glutathione *S*-transferase (GST) fusion proteins of PRMT1 and PRMT2 form a complex that can augment PRMT1 activity towards histone H4 (34). We sought to further expand this observation by pursuing how PRMT2 influences PRMT1 methylation of core histones either individually, in complex as calf thymus histones, recombinant human histone octamers, or in unmodified mononucleosomes. Zheng et al. demonstrated that individual PRMT enzymes have differing substrate specificity towards free histones, histone octamers, and nucleosomes (43). While individual PRMTs were unable to methylate recombinant unmodified mononucleosomes *in vitro* in their study, Vedadi et al. recently showed that H4K20 monomethylation in nucleosomes facilitated *in vitro* H4R3 methylation by PRMT1 (58). We also encountered differences in methylation depending on the macromolecular state of histones. Purified calf thymus histones H3 and H4 have several ε-*N*-methyllysine and ε-*N*-acetyllysine modifications in the N terminus of both histone proteins [H3K9(Ac), H3K14(Ac), H3K23(Ac), H3K27(Me), H4K16(Ac), H4K20(Me)], but not histone H2A (59, 60, 61). These modifications do not exist on the recombinantly expressed histones, histone octamers, or nucleosomes and could be a major contributing factor to the discrepancies in observed methylation levels for PRMT1/2 (62, 63). Additionally, arginine methylation may occur prior to histone oligomerization or nucleosome formation, which could also explain why we see methylation pattern differences among the substrates tested. We speculate that further epigenetic modifications could further enhance the activity of the PRMT1/2 complex towards mononucleosomes both *in vitro* and *in vivo*. Nevertheless, we showed that combining these two PRMTs resulted in altered substrate specificity for PRMT1.

### PRMT2 and the histone code

PRMT2 is capable of augmenting PRMT1 and PRMT6 activity towards histone H2A depending on the context in which the substrate is presented. We showed that histone H2AR3 is the primary site for PRMT1/2 (Fig. 5*E*). Another site for PRMT1 is H2AR11 (39, 64), whose methylation appeared to be modestly reduced by the H2AR11K mutation (Fig. 5*E*). Histone H2A sites for PRMT6 are reported as H2AR3 and H2AR29 (64, 65). Since H2AR29me2a is enriched at genes repressed by PRMT6, we speculate that increased PRMT2 expression in cells may increase PRMT6-specific gene repression *via* increased H2AR29 methylation by a PRMT2/6 complex (39, 66). We demonstrate through DSF that PRMT2 is capable of binding the H2A peptide, in a manner we hypothesize is similar to how Mona Gads SH3 binds the SLP-76 peptide (Fig. S22) (52). Given our results herein, it is possible that PRMT2 can modulate PRMT1 and PRMT6 activities *in vivo* towards H2A, resulting in substrate hypermethylation driving oncogenic expression. Further characterization is required to unravel the link between PRMT2 expression and H2A methylation *in vivo*.

In addition to histone H2A methylation, we provide *in vitro* evidence for PRMT2 altering PRMT1’s ability to methylate histone H3 (Fig. 5*B*). PRMT2 has been implicated either directly or indirectly with H3R8 methylation (12, 13, 14). It is possible that there may be conditions that enhance the catalytic activity of PRMT2 such that it can methylate H3R8 in a biologically relevant timeframe, but these conditions have yet to be identified and replicated *in vitro*. Our results provide an alternative mechanism for H3R8 methylation as being deposited by a more active PRMT enzyme (*e.g.*, PRMT1, PRMT6, or CARM1) whose activity is redirected by PRMT2 (34, 47, 48, 67).

Beyond the core histones, the combination of PRMT2/4 methylated a band in calf thymus histones consistent with the gel migration of linker histone H1 (Fig. 6*B*); however, we were unable to methylate histone H1 as a recombinant solo substrate (data not shown). Histone H1 arginine methylation has yet to be reported. Of specific interest is the site H1R51, which can be citrullinated to regulate pluripotent activation (68). The methylation of this arginine could theoretically result in inhibition of citrullination and in turn inhibit cell pluripotency. It will be important to show that histone H1 is methylated in cells before there is any further speculation.

### Concluding remarks

In this study, we demonstrated the influence of PRMT2 in redirecting PRMT1 substrate specificity towards histone H2A. These findings suggest that direct arginine methylation may not be PRMT2’s primary function, but instead it may serve as an adapter protein designed to mediate protein–protein interactions and recruit more active PRMTs for targeted substrate methylation. The PRMT2-dependent epigenetic modifications of histone proteins appear to rely on their macromolecular organization and quite possibly their modification status. Delving deeper into heteromeric PRMT2 complex activities will be imperative to understanding their biological implications and involvement with diseases like glioblastoma multiforme, hepatocellular carcinoma, and renal cell carcinoma.

## Experimental procedures

### Materials

SAM, SAH, and lyophilized calf thymus histones were purchased from Millipore Sigma (A7007, A9383, and H9250, respectively) and dissolved in 0.5 mM HCl to make 2 mM stock (SAM, SAH) or water to make 0.6 mg/ml stocks (histones). Radiolabeled cofactor *S*-adenosyl-L-[*methyl*-^14^C]-methionine (^14^C-SAM; 58 mCi mmol^−1^) was purchased from PerkinElmer (NEC363050UC). The H4 (Ac-SGRGKGGKGLGKGGAKR) peptide was purchased from Canada Peptide and was synthesized with N-terminal acetylation. The H3 (Ac-ARTKQTARKSTGGKAPRKQLA) and H2A (Ac-SGRGKQGGKARAKAKTRSSR) peptides were purchased from Biomatik and synthesized with N-terminal acetylation. Peptides were dissolved in water to prepare 2 mM stocks. Recombinant human H3.3 mononucleosomes and H3.3 octamers were purchased from EpiCypher (16-0012 and 16-8012, respectively). SYPRO Orange Protein Gel Stain was purchased from ThermoFisher (S-6650) as a 5000× concentrate in dimethylsulfoxide and was diluted to a 50× stock in water immediately before use. *NativeMark* unstained protein standards were purchased from Invitrogen (LC0725).

### DNA constructs

Cloning of PRMT1 (UnitProt ID: Q99873-3), PRMT1E153Q, PRMT2 (UnitProt ID: P55345-1), PRMT2E220Q, mCer-PRMT1, and mCit-PRMT2 in pET28a(+) (Novogene) have been previously described (34, 69). Cloning of PRMT2 Δ SH3 (aa 83–433) in pGEX-2T (Novogene) has been previously described (8). PRMT2H112Q and the PRMT2 SH3 domain were generated by mutagenesis (Quick Change Mutagenesis Kit, Agilent) on the PRMT2-pET28a(+) template using either 5′- CGT GGC AGG ATG AAG AGT ACT TCT AAG GCA GCT ATG GAA CTC TG-3′ and 5′- CAG AGT TCC ATA GCT GCC TTA GAA GTA CTC TTC ATC CTG CCA CG-3′ primers for PRMT2 SH3 that inserted a stop codon after the SH3 domain (aa 1–103) or 5′- AGCTATGGAACTCTGAAACTCCAGTTGGAGATGTTGGCAGACCAG-3′ and 5′- CTGGTCTGCCAACATCTCCAACTGGAGTTTCAGAGTTCCATAGCT-3′ for PRMT2H112Q. All other PRMT enzymes and histone plasmid information and cloning strategies can be found in Supporting information.

### Protein expression and purification

All PRMT2 constructs were transformed and expressed in *Escherichia coli* Arctic Express (DE3) (Stratagene), except for GST-PRMT2 Δ SH3, which was expressed in *E. coli* BL21 DE3 pLysS. All other protein constructs were transformed in *E. coli* Rosetta (DE3) (Stratagene) (Supporting information). In brief, cells were grown in LB media to an A_600_ of 0.6 and protein expression was induced with 1 mM IPTG at 16 °C for 16 h. Cells were harvested *via* centrifugation (10000*g*, 4 °C, 15 min) and cell pellets were frozen at −80 °C.

His-tagged PRMT purification followed the steps below, while histone and GST-PRMT purification is detailed in Supporting information. Native histone purification is detailed in Supporting information. Cell pellets containing hexa-histidine–tagged proteins were thawed on ice in a lysis buffer [2 ml/g wet weight of cells; 50 mM Hepes–KOH pH 7.4, 1 M NH_4_Cl, 10 mM MgCl_2_, 10 mM imidazole, 0.1% Triton X-100, 0.1% lysozyme, 25 U mL^-1^ DNAse 1, 1.0 mM PMSF, 7 mM β-mercaptoethanol (BME), and 1.0 mM EDTA-free protease inhibitor cocktail] and incubated for 1 h at 25 °C before further lysis *via* freeze-thaw. Homogenized cell lysates were immersed in liquid nitrogen until frozen, followed by immersion in a 25 °C water bath until fully thawed and repeated for a total of three cycles. Soluble proteins were separated by centrifugation (35000*g*, 4 °C, 1 h) and filtered through a 0.22 μM low protein-binding polyvinylidene fluoride membrane (Millex). Clarified lysates containing hexa-histidine tags were applied to pre-equilibrated Ni-NTA resin (GE healthcare) in wash buffer (50 mM Hepes–KOH pH 7.6, 1 M NH_4_Cl, 10 mM MgCl_2_, 10 mM BME, 10 mM imidazole, 1 mM PMSF) and eluted using stepwise imidazole elution buffers (same composition as wash buffer except for 100–400 mM imidazole). Fractions containing the protein (confirmed by 10% SDS-PAGE) were applied to a pre-equilibrated HiLoad26/600 Superdex 200 pg size-exclusion column in wash buffer (50 mM Tris–HCl, 100 mM NaCl, 1 mM DTT, pH 8.0). Eluted fractions containing the desired protein (evaluated by 10% SDS-PAGE analysis) were pooled and concentrated in a storage buffer [hexa-histidine–tagged proteins: 100 mM Hepes–KOH pH 8.0, 200 mM NaCl, 1 mM DTT, 10% glycerol, 2 mM EDTA; GST-tagged proteins: 50 mM Tris–HCl pH 7.5; 500 mM NaCl; 1 mM DTT, 10% glycerol (v/v)] using Amicon Ultra 15 ml filters [30-kDa molecular weight cut-off (Millipore)]. The fluorescently tagged proteins were quantified spectrophotometrically according to the extinction coefficient of the fluorophore: mCit (ε_516__nm_ = 77,000 M^-1^ cm^-1^) and mCer (ε_434 nm_ = 43,000 M^-1^ cm^-1^). All other protein concentrations were quantified by the Bradford assay (Quick Start Bradford Protein Assay; BioRad).

### Differential scanning fluorimetry

Reactions were prepared in methylation buffer (50 mM Hepes, pH 8.0, 10 mM NaCl, 1.0 mM DTT) with 7.5× SYPRO Orange to a final volume of 20 μl in a MicroAmp Fast Optical 96-well reaction plate (Applied Biosystems 4346907). Unless otherwise noted, 5 μM PRMT2, PRMT2 Δ SH3, or PRMT2H112Q was used, and all reactions were performed in triplicate. To determine effect of peptide and cofactor on PRMT2 stability, reactions were prepared with SAH, H2A, H3, or H4 peptide from 0 to 500 μM in triplicate. To determine heteromeric protein stabilization effects of PRMT1 towards PRMT2, PRMT2 Δ SH3, or PRMT2H112Q, either 1, 5, or 10 μM PRMT1 were incubated with 1, 5, or 10 μM PRMT2 (except for PRMT2H112Q and PRMT2 Δ SH3 where only 5 μM were tested). Prepared reaction plates were sealed with an optical adhesive cover (4360954) and incubated in the dark for 30 min at room temperature. Sealed plates were briefly centrifuged at 500*g* using an MPS 100 Microplate Spinner (Labnet). Plates were heated from 25 to 80 °C at a rate of 0.5 °C/min and analyzed using the StepOnePlus Real-Time PCR Systems (Applied Biosystems). Samples were excited at 490 nm and emission was measured at 580 nm after 30-s intervals. Data were plotted and analyzed in GraphPad Prism 9.5 (https://www.graphpad.com).

To evaluate PRMT melting stability, first-derivative analysis was performed with second-order smoothing for data presentation with GraphPad Prism 9.5. T_m_ values were derived from generating the second derivative of the raw fluorescence data (no smoothing) and calculating the x-intercept value (using Microsoft Excel 2023) that corresponds to the local maximum on the first-derivative curve. Statistical significance was measured using an ordinary one-way ANOVA test with Bonferroni’s multiple comparison correction applied. A statistical level of significance cut off <0.05 was used.

To evaluate PRMT2 Δ SH3 T_m_, the raw data were normalized as previously described (37). The normalized data were fit to a Boltzmann Sigmoidal curve using (Equation 1) from which the T_m_ could be generated.

(1) $$y=Bottom+\frac{\left(Top-Bottom\right)}{1+e^{(\frac{T_{m}-x}{Slope})}}$$

K_D_ values for specific ligands were generated by plotting T_m_ values against the concentration of ligand as a dose-response curve. The curve was fit to a single site ligand-binding model using (Equation 2) where x is the ligand concentration, y is the T_m_, and P is the protein concentration.

(2) $$y=Bottom+\left(Top-Bottom\right)\left(1-\frac{P-K_{D}-x+\sqrt{{\left(P+x+K_{D}\right)}^{2}-4Px}}{2P}\right)$$

### Native PAGE

PRMT enzymes (1 μM each) were mixed with methylation buffer and incubated at 37 °C for 1 h before being mixed with native PAGE electrophoresis loading buffer (100 mM Tris–Cl pH 6.8, 10% glycerol, 1.5% BME, 0.02% bromophenol blue) and applied to a 4 to 20% gradient PAGE gel. Gels were run with 5 μl *NativeMark* unstained protein standards for molecular weight estimation. The unstained gels were analyzed for absorbance at 365 nm using a BioRad Gel Doc XR imager using trans-UV setting with 0.1-s exposure (to detect mCer). Fluorescence of mCit-tagged proteins was detected by exciting the unstained gels at 488 nm and measuring emission at 520 nm using a Sapphire Biomolecular Imager. Proteins were stained and destained as described above.

### Histone methylation kinetics

PRMT1 (100 nM) was pre-incubated with PRMT2 (0, 100, 1000, or 10,000 nM) at 37 °C for 1 h before addition of 0 to 25 μM histone H2A or H3 and 10 μM ^14^C-SAM for an additional incubation at 37 °C for 2 h. The reactions were spotted (2 × 2 μl) in triplicate on P81 filter paper *via* dot-blot apparatus under vacuum. The filter paper was washed, stained, and radioactivity quantified by filter binding and phosphor screening (42). The relative phosphor signals were plotted against H2A, H3, or H4 protein concentrations, and the data were fit to either a simple linear regression model or the Michaelis–Menten (Equation 3) using Graph Pad Prism 9.5.0 to derive apparent values K_M (App)_ and *k*_cat (App)_.

(3) $$v=\frac{k_{cat\;\left(App\right)}{\left[E\right]}_{T}\left[S\right]}{K_{M\;\left(App\right)}+\left[S\right]}$$

### Gel radiography

Unless indicated otherwise, PRMT2 (or PRMT2 variant) was titrated from 10 to 10,000 nM against 2 μM of either PRMT1, 3, 4, 5 (*Caenorhabditis elegans* PRMT5, incubations at 25 °C), 6, 7, or 8 in methylation buffer and incubated for 1 h at 37 °C to allow for complexation. The methylation reactions were initiated by addition of 0.6 mg/ml calf thymus histones and 10 μM ^14^C-SAM and further incubated an additional 2 h. The reactions were quenched by addition of tricine-SDS loading dye (50 mM Tris–Cl pH 6.8, 0.32% SDS, 13% glycerol, 0.4% BME, 0.02% bromophenol blue) and boiled at 95 °C for 15 min. Proteins were then separated on Tris/Tricine/SDS 16.5% discontinuous PAGE gels. Proteins were stained, destained (described above), and dried down before exposure to a phosphor screen (exposure times are indicated in figure legends). Phosphor screens were analyzed by phosphor imaging using a Sapphire Biomolecular Imager. Densitometry analysis was performed by Image Studio Lite Ver 5.2, as described previously (42) to generate relative changes in histone methylation.

## Data availability

All data from this study can be found in this article or supporting information.

## Supporting information

This article contains supporting information (34, 70, 71).

## Conflict of interest

The authors declare that they have no conflicts of interest with the contents of this article.
