# Supplementary material for: Protein arginine N-methyltransferase 2 plays a noncatalytic role in the histone methylation activity of PRMT1
Source: J Biol Chem. 2023 Oct 19;299(12):105360. doi: 10.1016/j.jbc.2023.105360 (PMC10692916; doi:10.1016/j.jbc.2023.105360)
Supplement: Supporting information [file mmc1.pdf]

Supporting Information

Protein arginine N-methyltransferase 2 (PRMT2) plays a noncatalytic role in the histone methylation activity of PRMT1

Michael J. Rowley,[1] Riley A. Prout-Holm,[1] Rui Wen Liu,[1] Thordur Hendrickson-Rebizant,[2] Olufola O. Ige,[2] Ted M. Lakowski,[2] Adam Frankel[1]*

[1]Faculty of Pharmaceutical Sciences, The University of British Columbia, 2405 Wesbrook Mall, Vancouver, British Columbia V6T 1Z3, Canada

[2]College of Pharmacy, University of Manitoba, 750 McDermot Avenue West, Winnipeg, Manitoba R3E 0T5, Canada

*Corresponding author email address: adam.frankel@ubc.ca

Table of Contents

Supporting Information Methods……………………………………..…………………S-2

Supporting Information Figures……………………………………………………… S-4

Supporting Information Tables…………………………………………………………S-23

Supporting Information Methods

Plasmids. Pet28a_Synthetic_Human_H2A.1 (UniProt ID: P0C0S8) and Pet28a_Human_H3.3 (UniProt ID: P84243) were gifts from Joe Landry (Addgene plasmids # 42634 and # 42632, respectively). pMMTH+H1.4 (UniProt ID: A3R0T8) plasmid was a gift from Michael Rossen (Addgene plasmid # 157796). cePRMT5 in pET21a(+), and PRMT3, PRMT7, and PRMT8 in pGEX-2T were generous gifts from Rui-Ming Xu (Chinese Academy of Sciences) and Steven Clarke (UCLA), respectively. Human CARM1 in pET28a(+) has been described elsewhere (1). pET3+H3 was a gift from Dr. Karolin Luger (Colorado State University). Histone H2A mutants were generated by mutagenesis (Quick Change Mutagenesis Kit, Agilent) on the Pet28a_Synthetic_Human_H2A.1 (+) template using: 5' – GAG ATA TAC CAT GTC TGG TAA GGG TAA ACA AGG TGG TAA AGC C – 3' and 5' – GGC TTT ACC ACC TTG TTT ACC CTT ACC AGA CAT GGT ATA TCT C – 3' primers for H2AR3K; 5' – GTG GTA AAC AAG GTG GTA AAG CCA AGG CCA AAG CCA AAA CCC GCT – 3' and 5' – AGC GGG TTT TGG CTT TGG CCT TGG CTT TAC CAC CTT GTT TAC CAC – 3' primers for H2AR11K; 5' – GGT CTG CAA TTT CCG GTC GGT AAC GTT CAT CGT CTG CTG CGC AAA – 3' and 5' – TTT GCG CAG CAG ACG ATG AAC GTT ACC GAC CGG AAA TTG CAG ACC – 3' primers for H2AR29N. Histone H3 mutants were generated by mutagenesis (Quick Change Mutagenesis Kit, Agilent) on the Pet28a_Human_H3.3 template using: 5' - ACA AAG CAG ACT GCC GCC AAA TCG ACC GGT GGT – 3', and 5' - ACC ACC GGT CGA TTT GGC GGC AGT CTG CTT TGT – 3' primers for H3R8A.

Protein Expression. PRMT8 was transformed in *E. coli* BL21(DE3) pLysS (Stratagene). PRMT8 expression and cell harvest were done via the same protocol described for PRMT1 and PRMT2. Histone H2A was transformed into *E. coli* Arctic Express DE3 (Stratagene), whereas histones H3 and H4 were transformed in *E. coli* Rosetta DE3 (Stratagene). In brief, cells were grown in LB media to an OD_{600} of 0.6 and protein expression was induced with 1 mM isopropyl β-D-1 thiogalactopyranoside (IPTG) at 16 ºC for 16 h. Cells were harvested via centrifugation (10000 x *g*, 4 ºC, 15 min) and cell pellets were frozen at -80ºC.

Purification of GST-tagged Proteins. Cell pellets containing GST-tagged proteins (PRMT3, PRMT7, PRMT8, and PRMT2ΔSH3) were lysed using the same protocol as listed in the main text except using a PBS lysis buffer [2 mL/g pellet; 1x PBS pH 7.3, 5% glycerol; 1 mM EDTA; 1 mM EGTA, 0.1% Triton X-100, 0.1% lysozyme, 25 U mL^{-1} DNAse 1, 1.0 mM phenylmethanesulphonyl fluoride (PMSF), 7 mM β-mercaptoethanol (BME), and 1.0 mM EDTA-free protease inhibitor cocktail (PIC)]. Following separation of soluble proteins, GST-tagged proteins were injected to a pre-equilibrated 5 mL GST-Trap FF column (GE healthcare) (pre-equilibrated in PBS pH 7.3 and 1 mM DTT) and eluted with reduced glutathione (50 mM Tris-HCl, 10 mM reduced glutathione, 1 mM DTT, pH 8). Protein concentration and quantification was performed using same protocols described in main text.

The GST-tag was cleaved from PRMT2ΔSH3 by incubating the protein with thrombin in PBS pH 7.3 for 16 h. The uncleaved GST-tagged protein, free GST-tag, and thrombin was captured on the GST-Trap FF column and 1 mL Benzamidine FF column (GE healthcare) (pre-equilibrated in PBS pH 7.3, 1 mM DTT) and the cleaved protein was collected in the flow-through. The protein was concentrated and quantified as described in the main text.

Histone Purification. *X. Laevis* histone H4 (same amino acid sequence as human histone H4) was purified according to previously published methods (2), whereas human histones H2A and H3 were purified according to the rapid histone purification method (3). Cell pellets containing native histones (H2A and H3 constructs) were thawed on ice in a lysis buffer [5 mL/g pellet; 6 M Urea, 40 mM sodium acetate pH 5.2, 200 mM NaCl, 10 mM lysine, 0.1% Triton X-100, 1.0 mM PMSF, 5 mM BME, and 1.0 mM EDTA-free protease inhibitor cocktail (PIC)] and incubated for 1 h at 25 ºC before further lysis via freeze-thaw. Homogenized cell lysates were immersed in liquid nitrogen until frozen, followed by immersion in a 25 ºC water bath until fully thawed and repeated for a total of three cycles. Soluble proteins were separated by centrifugation (35000 x *g*, 4 ºC, 1 h) and filtered through a 0.22-µM low protein binding PVDF membrane (Millex). Clarified lysates were applied to pre-equilibrated QHP column coupled to an SP-HP column (GE healthcare) in wash buffer [6 M Urea, 40 mM sodium acetate pH 5.2, 10 mM lysine, 5 mM BME, 1 mM PMSF]. After the lysate was applied and 1 CV was passed, the QHP containing the DNA was removed and the column was washed with another 4 CV of wash buffer. The bound histones were eluted over a NaCl gradient (same composition as wash buffer except for 1 M NaCl). Protein fractions were separated on a 16.5% Tricine-SDS-PAGE to confirm which fraction contained the protein. Eluted proteins were placed in 5000-Da MWCO dialysis tubing and dialyzed against refolding buffer [100 mM HEPES-KOH pH 8.0, 2 M NaCl, 1 mM BME, 1 mM EDTA] over 12 h at 4 ºC. The dialysate was replaced twice to ensure removal of all salts. Histones were quantified by spectrophotometry according to the extinction coefficient, H2A and H3.3 ε_{280} = 4470 $M^{-1}cm^{-1}$.

References

1. Lakowski, T. M., ’t Hart, P., Ahern, C. A., Martin, N. I., and Frankel, A. (2010) Nη-substituted arginyl peptide inhibitors of protein arginine N-methyltransferases. *ACS Chem. Biol.* **60**, 45–58

2. Pak, M. L., Lakowski, T. M., Thomas, D., Vhuiyan, M. I., Hüsecken, K., and Frankel, A. (2011) A protein arginine N -methyltransferase 1 (PRMT1) and 2 heteromeric interaction increases PRMT1 enzymatic activity. *Biochemistry*. **50**, 8226–8240

3. Klinker, H., Haas, C., Harrer, N., Becker, P. B., and Mueller-Planitz, F. (2014) Rapid purification of recombinant histones. *PLoS One*.

Supporting Information Figures

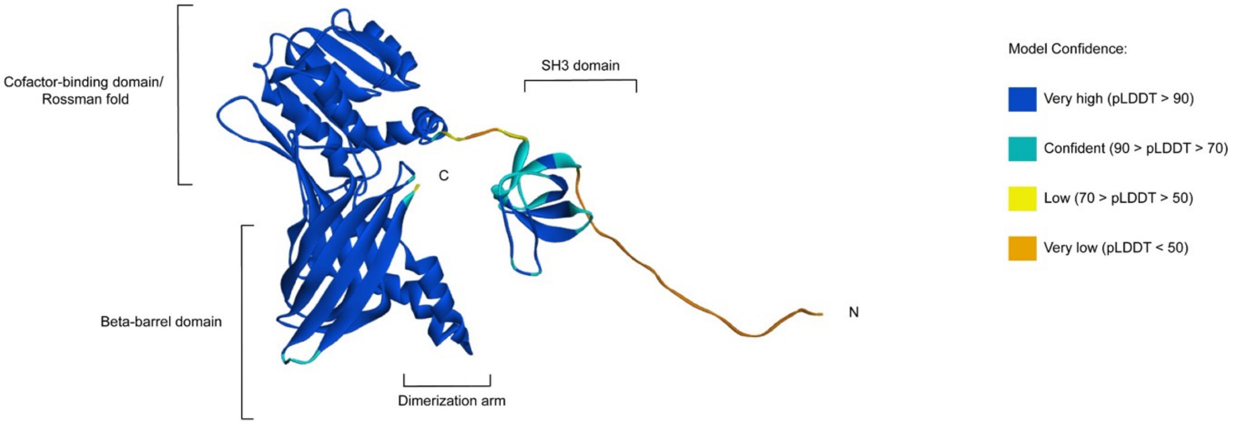

Supporting Figure 1. Predicted structure of full-length PRMT2. Model of the full-length human PRMT2(1-433) structure with colors showing model confidence by AlphaFold. Images rendered by Biovia Discovery Studio 2021.

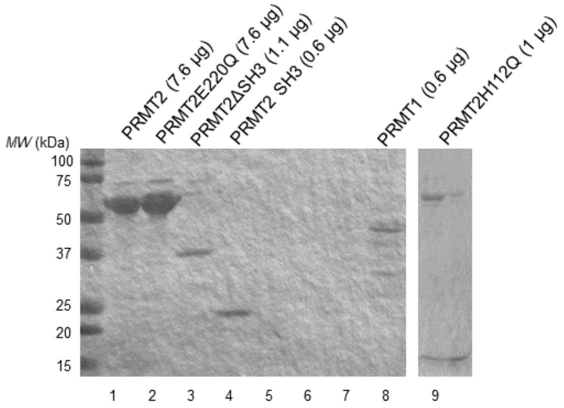

Supporting Figure 2. Purity of recombinant PRMT enzymes used in this study. Enzymes used in this study separated on 10% SDS-PAGE and stained by Coomassie Brilliant Blue.

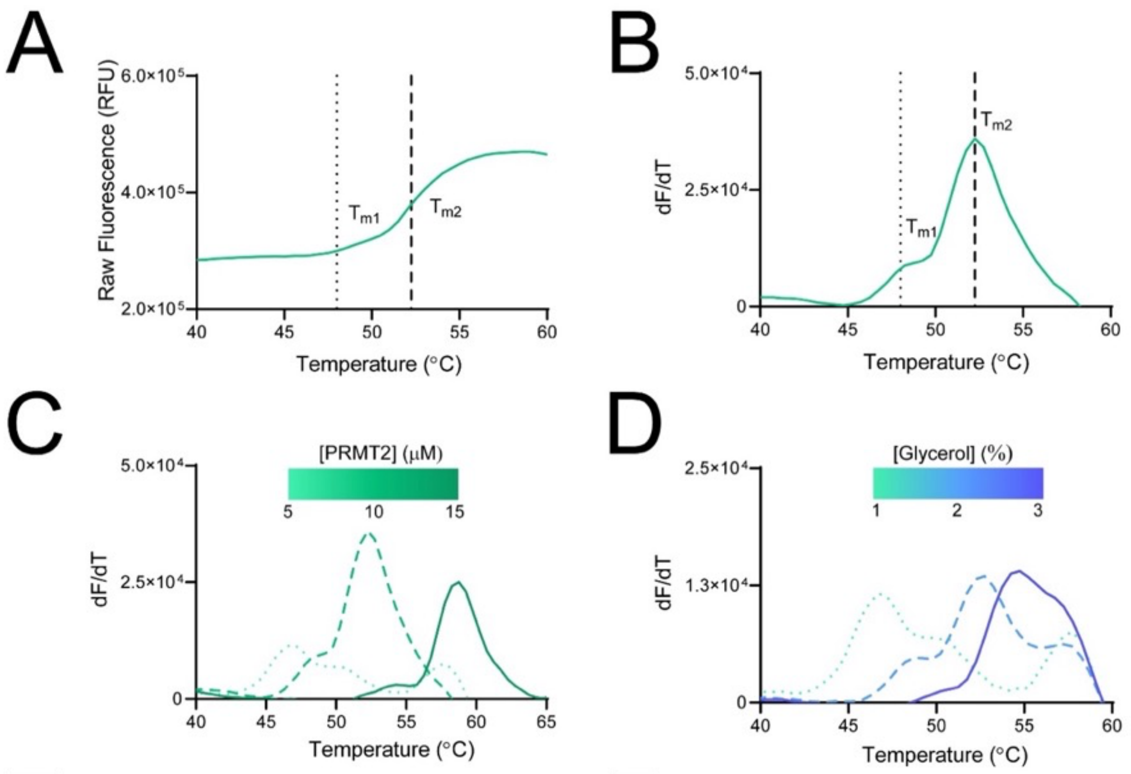

Supporting Figure 3. PRMT2 thermal stability measured by DSF. Thermal melt of 10 µM PRMT2 in DSF showing (A) averaged raw fluorescence (in relative florescence units (RFU)) and (B) first derivative data (in relative florescence units per second (RFU/s)). Evaluation of PRMT2 thermal stability at (C) different enzyme concentrations, and (D) in presence of increasing concentrations of glycerol at 5 µM PRMT2 (n = 3). Raw fluorescence spectra can be found in **Supporting Figure 4.** Tabulated melting temperatures and statistical analysis available in **Supporting Tables 1** and **2**.

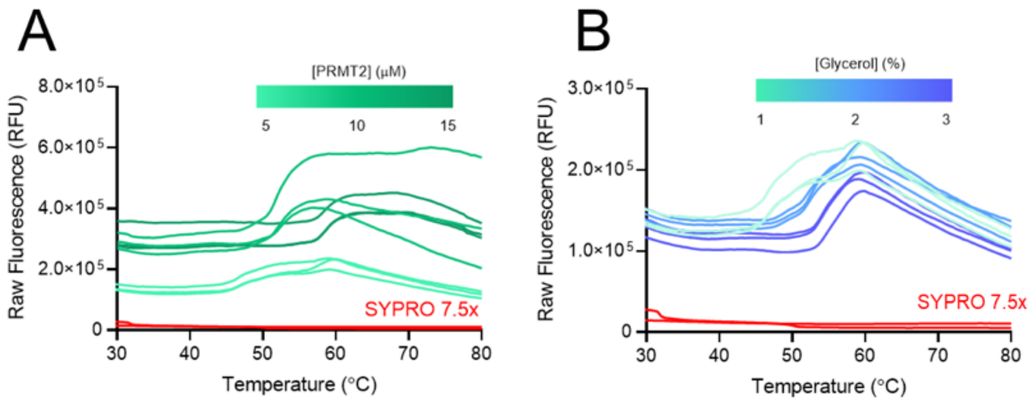

Supporting Figure 4. Thermal melt of PRMT2 in DSF. (A) Raw fluorescence from 7.5x SYPRO^{TM} Orange binding to three different concentrations of PRMT2 over a broad temperature range, and (B) the corresponding amount of glycerol at each protein concentration added to 5 µM PRMT2 (n = 3). Dye-only control included in red (n = 2). Tabulated melting temperatures and statistical analysis available in **Supporting Tables 1** and **2**.

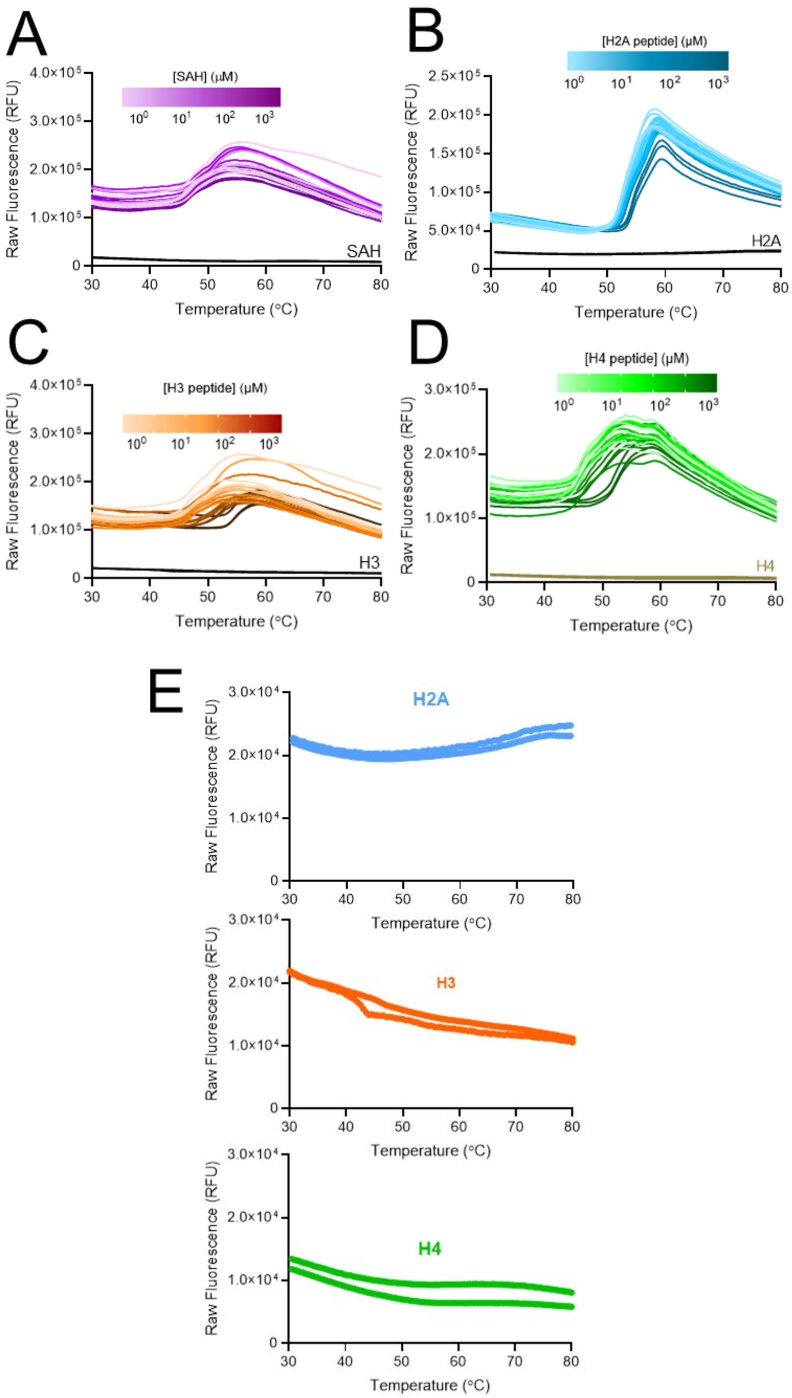

Supporting Figure 5. Impact of ligands on PRMT2 thermal stability. Raw fluorescence from 7.5x SYPRO™ Orange binding to PRMT2 (5 μM) over a broad temperature range in presence of increasing concentrations of (A) SAH (purple), (B) H2A peptide (blue), (C) H3 peptide (orange), or (D) H4 peptide (green) (n = 3). Peptide-only controls are shown in (E). Ligand-only controls included in each figure (n = 2). Tabulated melting temperatures and statistical analysis available in **Supporting Tables 3** and **4**.

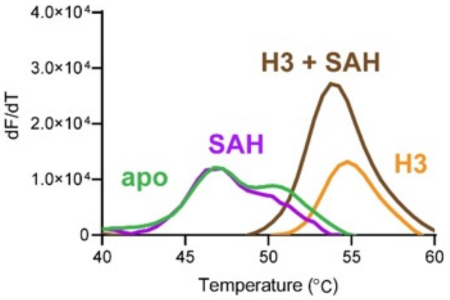

Supporting Figure 6. PRMT2 bisubstrate thermal stabilization analysis. PRMT2 (5 μM) was incubated with saturating amounts (500 μM) of SAH, and/or H3 peptide and the thermal stabilization of these ligands on PRMT2 was assessed by DSF. First-derivative melting traces (in relative florescence units per second (RFU/s)) are the average of three replicates; error bars show standard deviations. Tabulated melting temperatures and statistical analysis available in **Supporting Tables 5** and **6**.

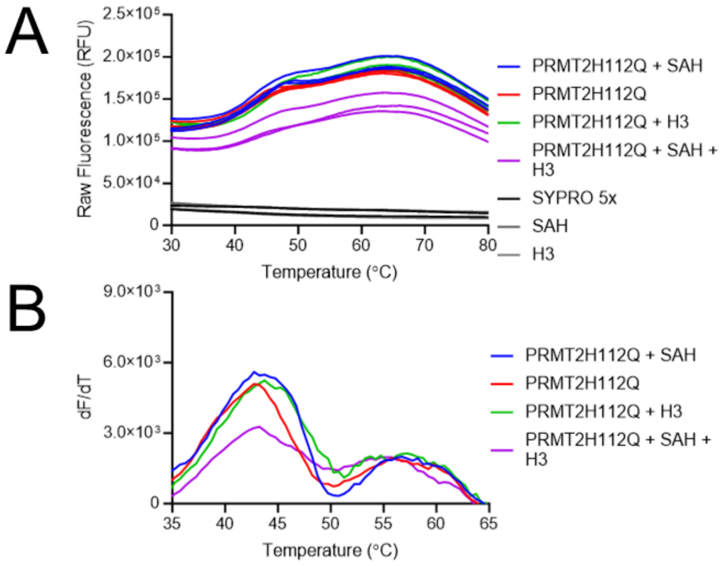

Supporting Figure 7. Thermal melt of PRMT2H112Q in DSF. (A) Raw fluorescence from 7.5x SYPRO^{TM} Orange binding to 5 μM PRMT2H112Q over a broad temperature range in presence or absence of saturating H3 peptide and/or SAH (500 μM). (B) First-derivative melting curves (in relative florescence units per second (RFU/s)) of PRMT2H112Q melt curves shown in A. Tabulated melting temperatures and statistical analysis available in **Supporting Tables 7** and **8**.

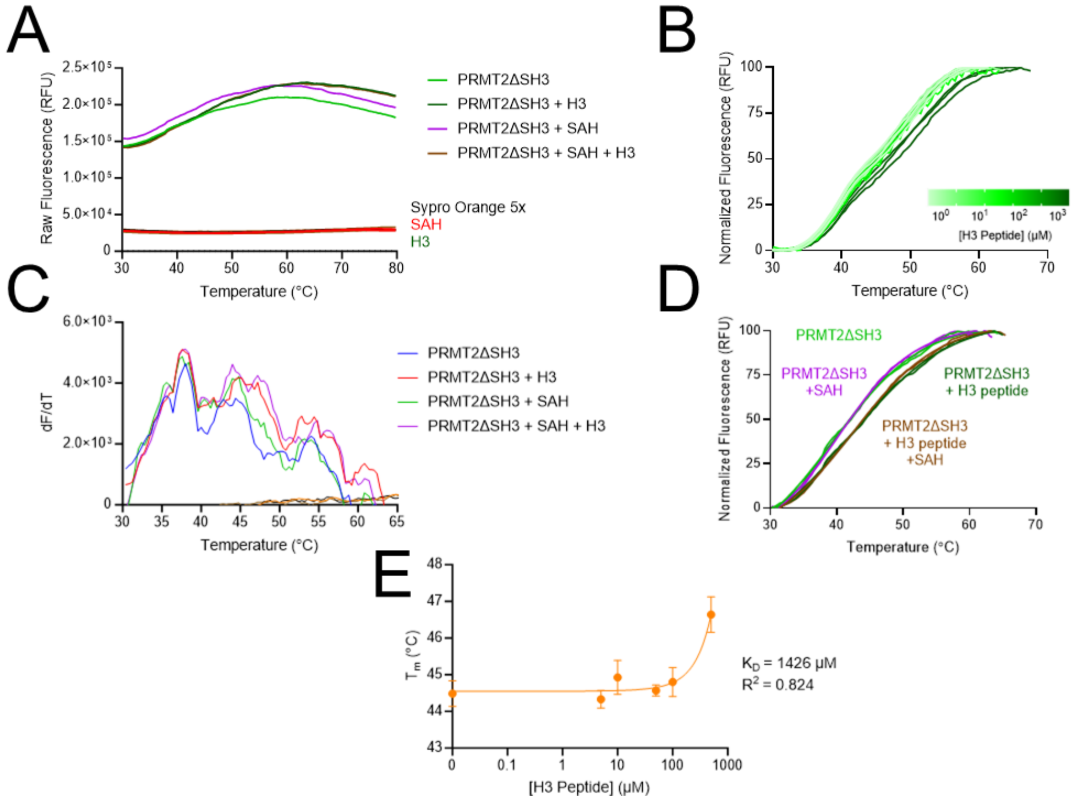

Supporting Figure 8. Thermal melt of PRMT2ΔSH3 in DSF. (A) Raw fluorescence from 7.5x SYPRO^{TM} Orange binding to 5 μM PRMT2ΔSH3 over a broad temperature range in presence or absence of saturating H3 peptide and/or SAH (500 μM). (B) Normalized fluorescence of SYPRO^{TM} Orange and PRMT2ΔSH3 with increasing concentration of H3 peptide (0-500 μM). (C) First-derivative melting curves (in relative florescence units per second (RFU/s)) of PRMT2 ΔSH3 melt curves shown in A. (D) Normalized fluorescence of SYPRO^{TM} Orange and PRMT2ΔSH3 in the presence or absence of SAH and/or H3 peptide (n = 3). (E) DSF-derived K_D for H3 peptide binding to PRMT2ΔSH3. Ligand-only controls included in (A). Tabulated melting temperatures and statistical analysis available in **Supporting Tables 9-11**.

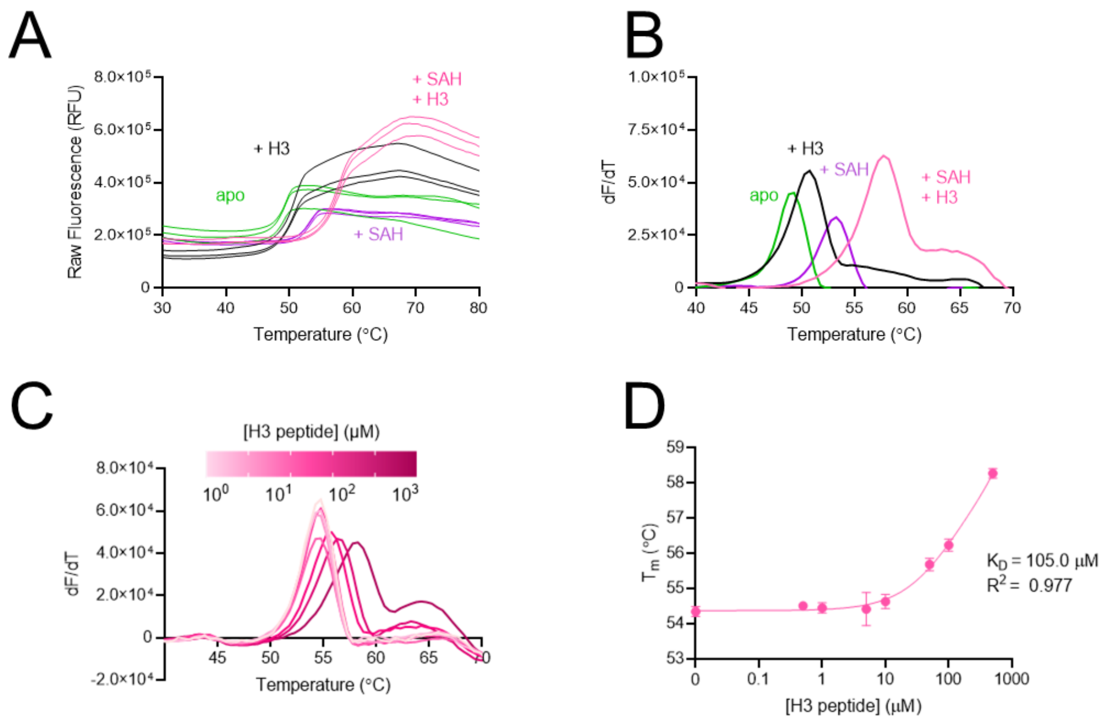

Supporting Figure 9. Impact of H3 peptide on PRMT1 thermal stability. (A) Raw fluorescence and (B) first-derivative data (in relative florescence units per second (RFU/s)) from 7.5x SYPRO^{TM} Orange binding to 5 µM PRMT1 over a broad temperature range in the presence or absence of saturating H3 peptide and/or SAH (500 µM). (C) First-derivative data (in relative florescence units per second (RFU/s)) from 5 µM PRMT1 in presence of increasing H3 peptide while holding SAH constant (500 µM) and (D) DSF-derived K_D for H3 peptide binding to PRMT1. Tabulated melting temperatures and statistical analysis available in **Supporting Tables 12-14**.

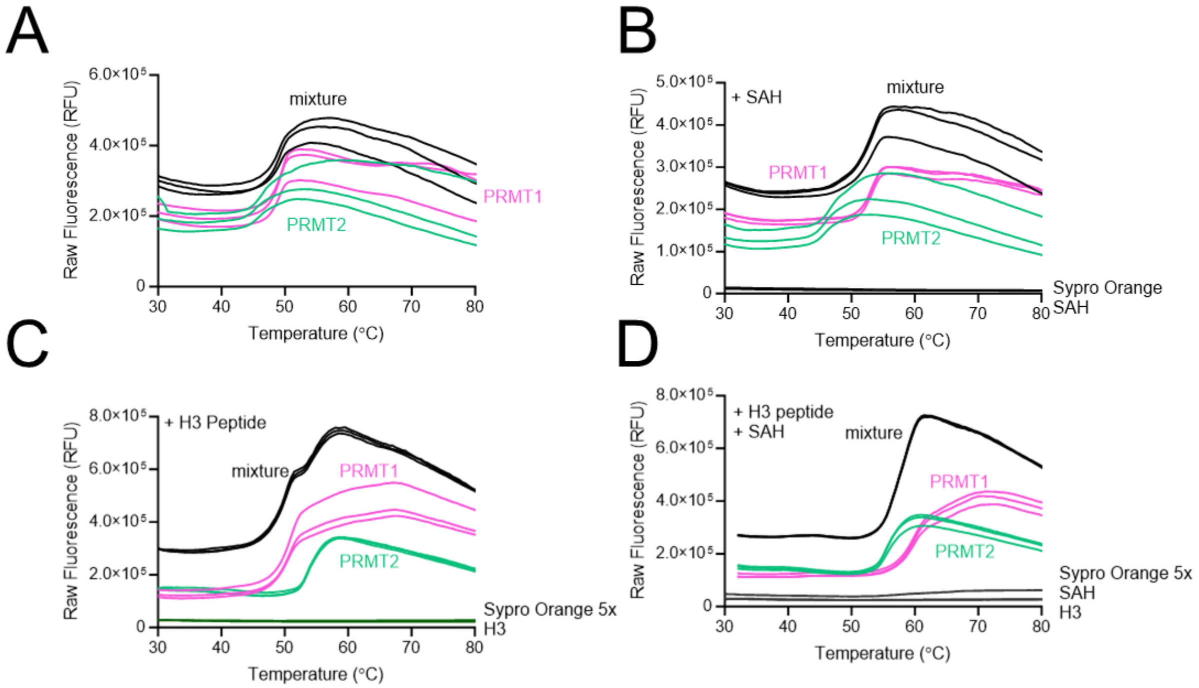

Supporting Figure 10. PRMT1/2 complex thermal stability. Raw fluorescence from 7.5x SYPRO^{TM} Orange binding to (A) 5 µM PRMT1 and 5 µM PRMT2 over a broad temperature range in absence of cofactor, (B) presence of SAH (500 µM), (C) H3 peptide (500 µM), or (D) both SAH and H3 peptide (500 µM) (n = 3). Ligand-only controls included in each panel (n = 2). Tabulated melting temperatures and statistical analysis available in **Supporting Tables 15** and **16**.

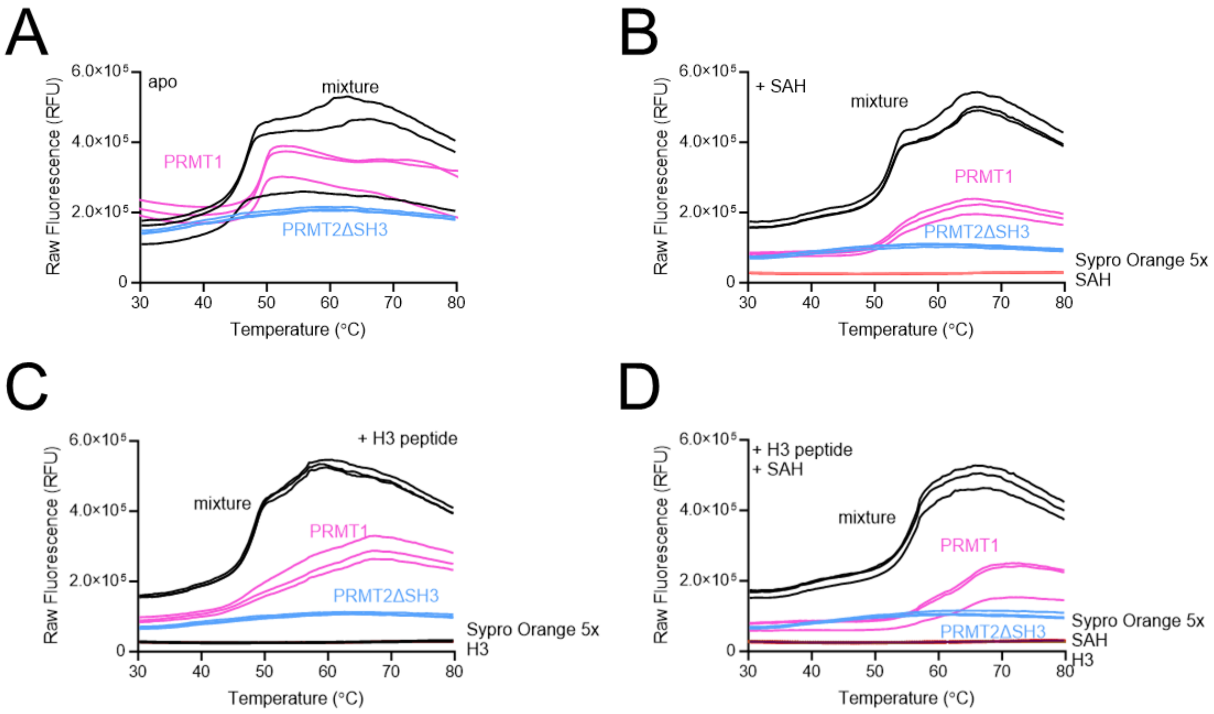

Supporting Figure 11. PRMT1/PRMT2ΔSH3 complex thermal stability. Raw fluorescence from 7.5x SYPRO™ Orange binding to 5 µM PRMT1 and 5 µM PRMT2ΔSH3 over a broad temperature range (A) in absence of ligands, (B) 500 µM SAH, (C) 500 µM H3 peptide, or (D) both SAH and H3 peptide at 500 µM each (n = 3). Ligand-only controls included in each figure (n = 2). Tabulated melting temperatures and statistical analysis available in **Supporting Tables 17** and **18**.

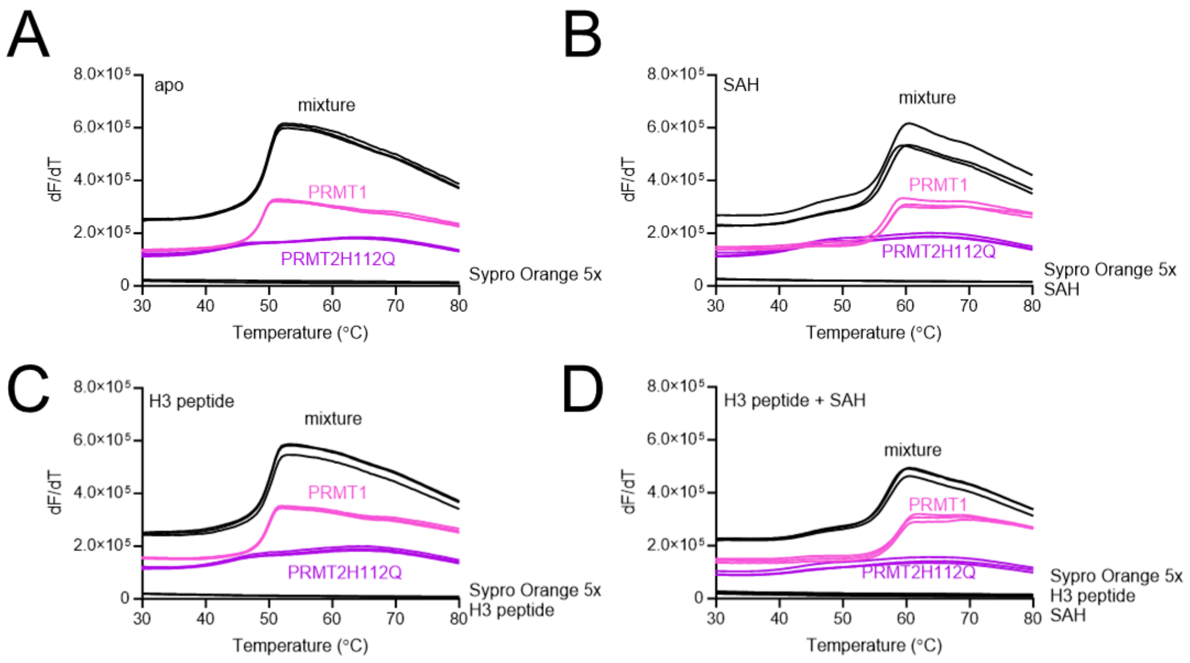

Supporting Figure 12. PRMT1/PRMT2H112Q complex thermal stability. Raw fluorescence from 7.5x SYPRO™ Orange binding to 5 µM PRMT1 and 5 µM PRMT2H112Q over a broad temperature range (A) in absence of ligands, (B) 500 µM SAH, (C) 500 µM H3 peptide, or (D) both SAH and H3 peptide at 500 µM each (n = 3). Ligand-only controls included in each figure (n = 2). Tabulated melting temperatures and statistical analysis available in **Supporting Tables 19** and **20**.

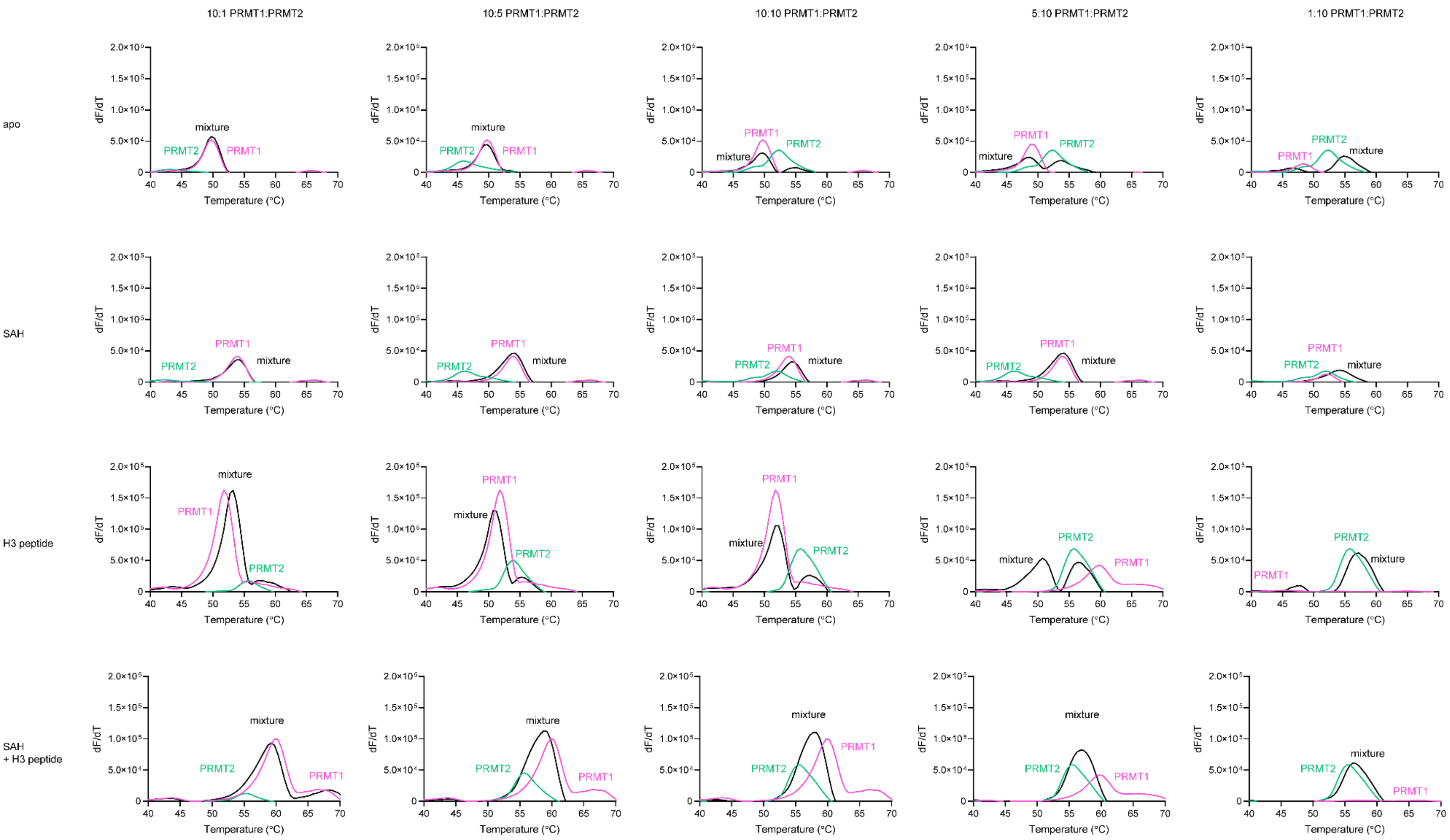

Supporting Figure 13. PRMT1/2 complex thermal stability varying protein concentrations. First-derivative of fluorescence with respect to temperature (in relative florescence units per second (RFU/s)) for 7.5x SYPRO™ Orange binding to 1-10 µM PRMT1 and 1-10 µM PRMT2 over a broad temperature range in absence of ligands, 500 µM SAH, 500 µM H3 peptide, or both SAH and H3 peptide at 500 µM each as indicated (n = 3). Tabulated melting temperatures available in **Supporting Table 21**.

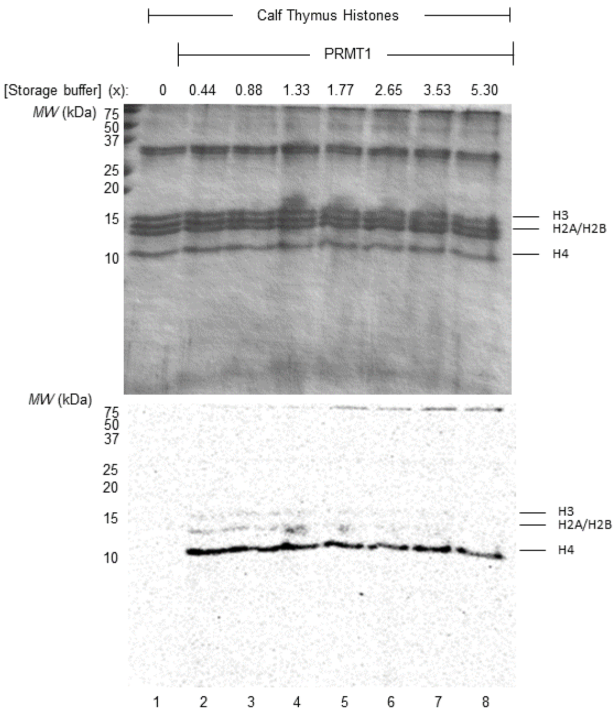

Supporting Figure 14. Impact of storage buffer on PRMT1 methylation of calf thymus histones. PRMT1 (1 µM) methylation reactions using 10 µM ^{14}C-SAM and 10 µg calf thymus histones with increasing amounts of storage buffer (1x =100 mM HEPES-KOH pH 8.0, 200 mM NaCl, 1 mM DTT, 10% glycerol, 2 mM EDTA). Reactions were separated on a 16.5% Tricine SDS-PAGE and visualized by Coomassie staining (top), followed by phosphor imaging analysis after 24-h exposure (bottom).

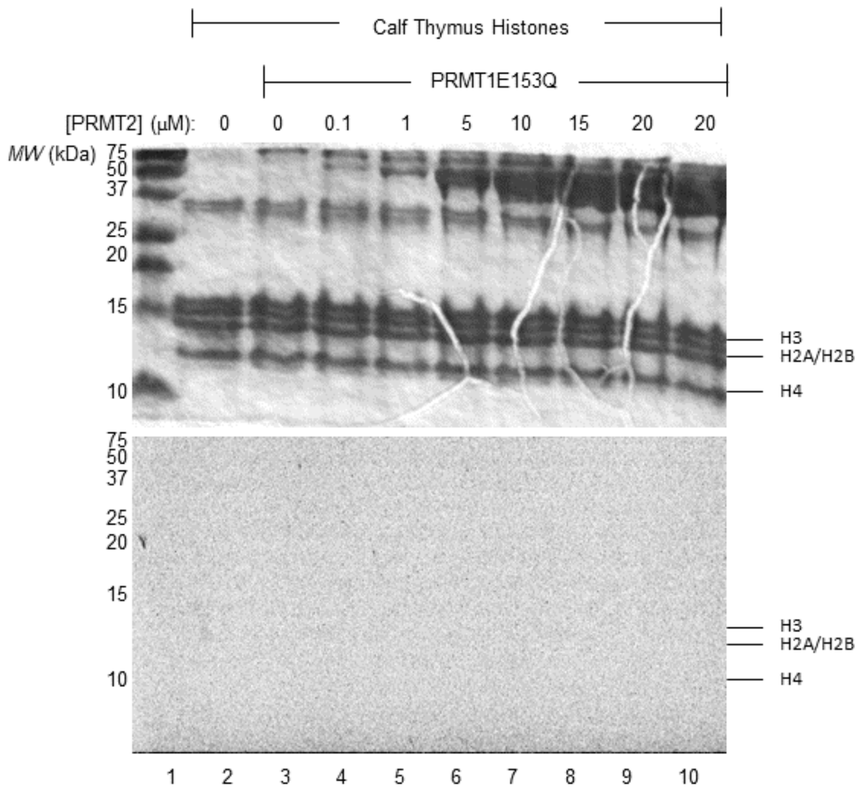

Supporting Figure 15. PRMT1E153Q/2 methylation of calf thymus histones. PRMT1E153Q (1 μM) methylation reactions using 10 μM ^{14}C-SAM and 10 μg calf thymus histones with increasing amounts of PRMT2. Reactions were separated on a 16.5% Tricine SDS-PAGE and visualized by Coomassie staining (top), followed by phosphor imaging analysis after 48-h exposure (bottom).

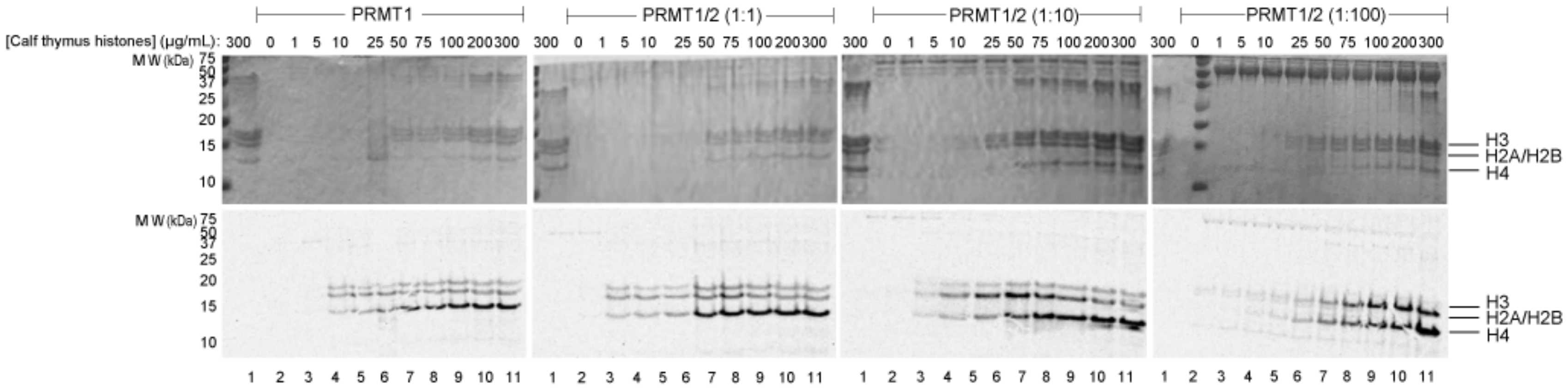

Supporting Figure 16. Gel radiography analysis of calf thymus histone methylation by PRMT1/2. PRMT1 (100 nM) was pre-incubated with PRMT2 (0, 100, 1000, 10000 nM) prior to reaction with 10 µM ^{14}C-SAM and 0-300 µg/mL calf thymus histones for 2 h. (A) Reactions were separated on 16.5% Tricine-SDS-PAGE, Coomassie stained (top) dried down and exposed to storage phosphor for 12 h before imaging (bottom). Tabulated values from the densitometry analysis can be found in **Supporting Table 22**.

BLOT A	1	2	3	4	5	6	7	8	9	10	11	12
A	H2A (25 μM)			PRMT1/2 (1:0)			PRMT1/2 (1:0) + 0.1 μM H2A			PRMT1/2 (1:0) + 0.5 μM H2A		
B	PRMT1/2 (1:0) + 1 μM H2A			PRMT1/2 (1:0) + 5 μM H2A			PRMT1/2 (1:0) + 10 μM H2A			PRMT1/2 (1:0) + 15 μM H2A		
C	PRMT1/2 (1:0) + 20 μM H2A			PRMT1/2 (1:0) + 25 μM H2A			PRMT1/2 (1:100)			PRMT1/2 (1:100) + 0.1 μM H2A		
D	PRMT1/2 (1:100) + 0.5 μM H2A			PRMT1/2 (1:100) + 1 μM H2A			PRMT1/2 (1:100) + 5 μM H2A			PRMT1/2 (1:100) + 10 μM H2A		
E	PRMT1/2 (1:100) + 15 μM H2A			PRMT1/2 (1:100) + 20 μM H2A			PRMT1/2 (1:100) + 25 μM H2A					
**BLOT B**	1	2	3	4	5	6	7	8	9	10	11	12
A	H2A (25 μM)			PRMT1/2 (1:1)			PRMT1/2 (1:1) + 0.1 μM H2A			PRMT1/2 (1:1) + 0.5 μM H2A		
B	PRMT1/2 (1:1) + 1 μM H2A			PRMT1/2 (1:1) + 5 μM H2A			PRMT1/2 (1:1) + 10 μM H2A			PRMT1/2 (1:1) + 15 μM H2A		
C	PRMT1/2 (1:1) + 20 μM H2A			PRMT1/2 (1:1) + 25 μM H2A			PRMT1/2 (1:10)			PRMT1/2 (1:10) + 0.1 μM H2A		
D	PRMT1/2 (1:10) + 0.5 μM H2A			PRMT1/2 (1:10) + 1 μM H2A			PRMT1/2 (1:10) + 5 μM H2A			PRMT1/2 (1:10) + 10 μM H2A		
E	PRMT1/2 (1:10) + 15 μM H2A			PRMT1/2 (1:10) + 20 μM H2A			PRMT1/2 (1:10) + 25 μM H2A					

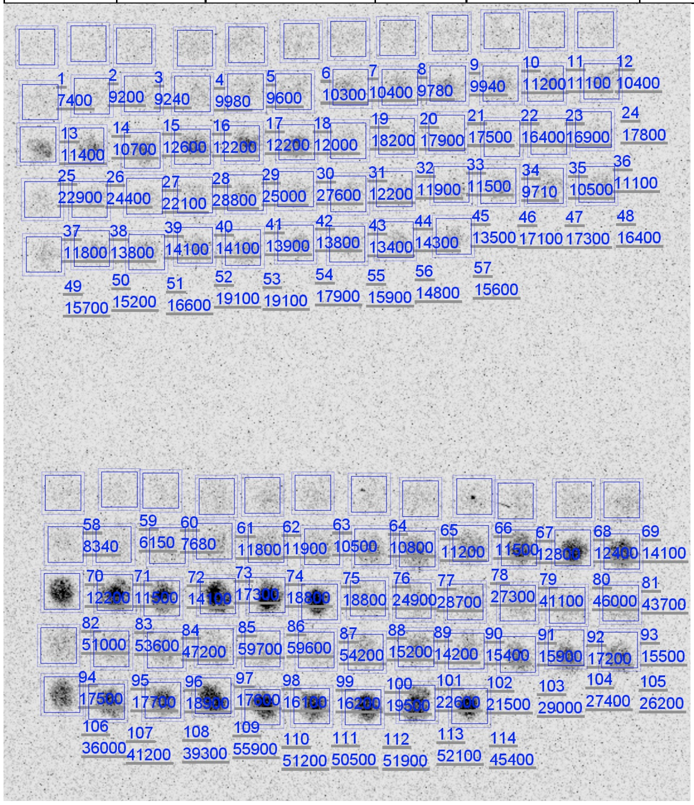

Supporting Figure 17. FBAPS analysis of H2A methylation by PRMT1/2. PRMT1 (100 nM) was pre-incubated with PRMT2 (0, 100, 1000, 10000 nM) prior to reaction with 10 μM ^{14}C-SAM and the indicated concentration of recombinant H2A for 2 h. Reactions were spotted on P81 filter paper (n = 3) according to grid (top) via dot-blot apparatus under vacuum, washed 5 times in 50 mM sodium phosphate pH 9.0, dried down and exposed to storage phosphor for 12 h before imaging (bottom). Tabulated densitometry data available in **Supporting Table 23**.

BLOT A	1	2	3	4	5	6	7	8	9	10	11	12
A	H3 (25 µM)			PRMT1/2 (1:0)			PRMT1/2 (1:0) + 0.1 µM H3			PRMT1/2 (1:0) + 0.5 µM H3		
B	PRMT1/2 (1:0) + 1 µM H3			PRMT1/2 (1:0) + 5 µM H3			PRMT1/2 (1:0) + 10 µM H3			PRMT1/2 (1:0) + 15 µM H3		
C	PRMT1/2 (1:0) + 20 µM H3			PRMT1/2 (1:0) + 25 µM H3			PRMT1/2 (1:100)			PRMT1/2 (1:100) + 0.1 µM H3		
D	PRMT1/2 (1:100) + 0.5 µM H3			PRMT1/2 (1:100) + 1 µM H3			PRMT1/2 (1:100) + 5 µM H3			PRMT1/2 (1:100) + 10 µM H3		
E	PRMT1/2 (1:100) + 15 µM H3			PRMT1/2 (1:100) + 20 µM H3			PRMT1/2 (1:100) + 25 µM H3					
**BLOT B**	1	2	3	4	5	6	7	8	9	10	11	12
A	H3 (25 µM)			PRMT1/2 (1:1)			PRMT1/2 (1:1) + 0.1 µM H3			PRMT1/2 (1:1) + 0.5 µM H3		
B	PRMT1/2 (1:1) + 1 µM H3			PRMT1/2 (1:1) + 5 µM H3			PRMT1/2 (1:1) + 10 µM H3			PRMT1/2 (1:1) + 15 µM H3		
C	PRMT1/2 (1:1) + 20 µM H3			PRMT1/2 (1:1) + 25 µM H3			PRMT1/2 (1:10)			PRMT1/2 (1:10) + 0.1 µM H3		
D	PRMT1/2 (1:10) + 0.5 µM H3			PRMT1/2 (1:10) + 1 µM H3			PRMT1/2 (1:10) + 5 µM H3			PRMT1/2 (1:10) + 10 µM H3		
E	PRMT1/2 (1:10) + 15 µM H3			PRMT1/2 (1:10) + 20 µM H3			PRMT1/2 (1:10) + 25 µM H3					

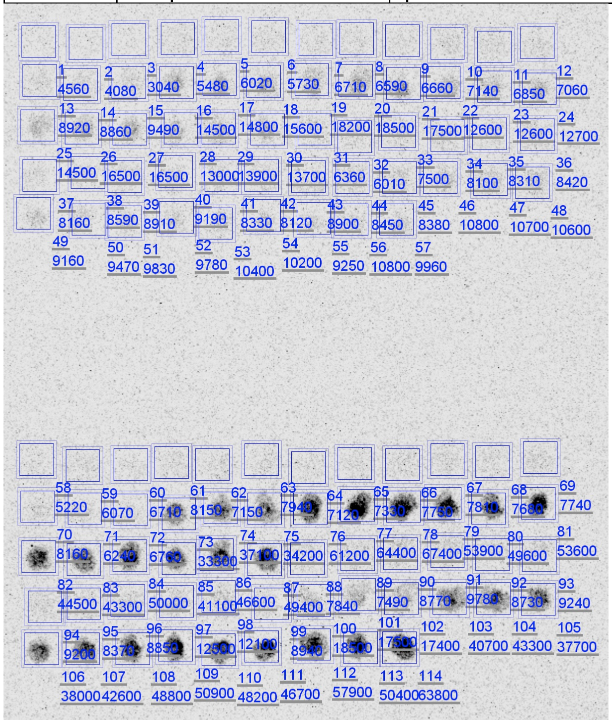

Supporting Figure 18. FBAPS analysis of H3 methylation by PRMT1/2. PRMT1 (100 nM) was pre-incubated with PRMT2 (0, 100, 1000, 10000 nM) prior to reaction with 10 µM ^{14}C-SAM and the indicated concentration of recombinant H3 for 2 h. Reactions were spotted on P81 filter paper (n = 3) according to grid (top) via dot-blot apparatus under vacuum, washed 5 times in 50 mM sodium phosphate pH 9.0, dried down and exposed to storage phosphor for 12 h before imaging (bottom). Tabulated densitometry data available in **Supporting Table 24**.

BLOT A	1	2	3	4	5	6	7	8	9	10	11	12
A	H4 (20 μM)			PRMT1/2 (1:0)			PRMT1/2 (1:0) + 0.1 μM H4			PRMT1/2 (1:0) + 0.5 μM H4		
B	PRMT1/2 (1:0) + 1 μM H4			PRMT1/2 (1:0) + 5 μM H4			PRMT1/2 (1:0) + 10 μM H4			PRMT1/2 (1:0) + 15 μM H4		
C	PRMT1/2 (1:0) + 20 μM H4			PRMT1/2 (1:1)			PRMT1/2 (1:1) + 0.1 μM H4			PRMT1/2 (1:1) + 0.5 μM H4		
D	PRMT1/2 (1:1) + 1 μM H4			PRMT1/2 (1:1) + 5 μM H4			PRMT1/2 (1:1) + 10 μM H4			PRMT1/2 (1:1) + 15 μM H4		
E	PRMT1/2 (1:1) + 20 μM H4			PRMT1/2 (1:10)			PRMT1/2 (1:10) + 0.1 μM H4			PRMT1/2 (1:10) + 0.5 μM H4		
F	PRMT1/2 (1:10) + 1 μM H4			PRMT1/2 (1:10) + 5 μM H4			PRMT1/2 (1:10) + 10 μM H4			PRMT1/2 (1:10) + 15 μM H4		
G	PRMT1/2 (1:10) + 20 μM H4			PRMT1/2 (1:100)			PRMT1/2 (1:100) + 0.1 μM H4			PRMT1/2 (1:100) + 0.5 μM H4		
H	PRMT1/2 (1:100) + 1 μM H4			PRMT1/2 (1:100) + 5 μM H4			PRMT1/2 (1:100) + 10 μM H4			PRMT1/2 (1:100) + 15 μM H4		
**BLOT B**	1	2	3	4	5	6	7	8	9	10	11	12
A	PRMT1/2 (1:100) + 20 μM H4											

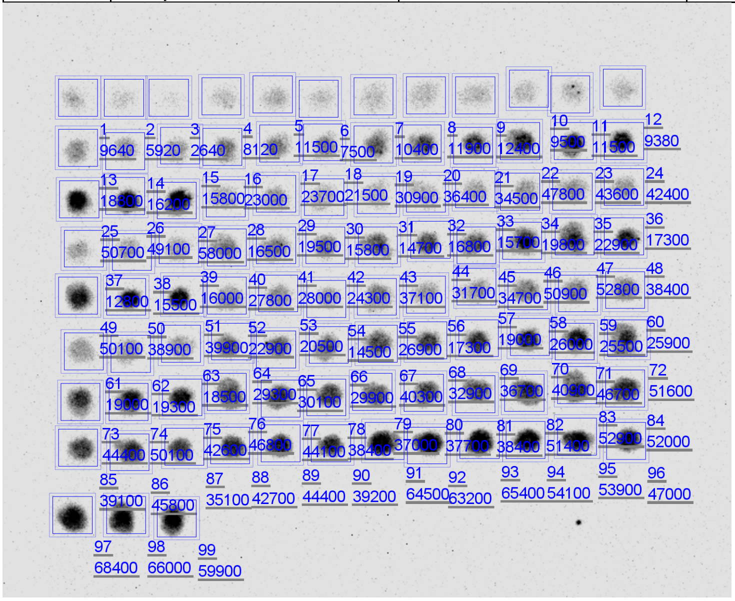

Supporting Figure 19. FBAPS analysis of H4 methylation by PRMT1/2. PRMT1 (100 nM) was pre-incubated with PRMT2 (0, 100, 1000, 10000 nM) prior to reaction with 10 μM ^{14}C-SAM and the indicated concentration of recombinant H3 for 2 h. Reactions were spotted on P81 filter paper (n = 3) according to grid (top) via dot-blot apparatus under vacuum, washed 5 times in 50 mM sodium phosphate pH 9.0, dried down and exposed to storage phosphor for 48 h before imaging (bottom). Tabulated densitometry data available in **Supporting Table 25**.

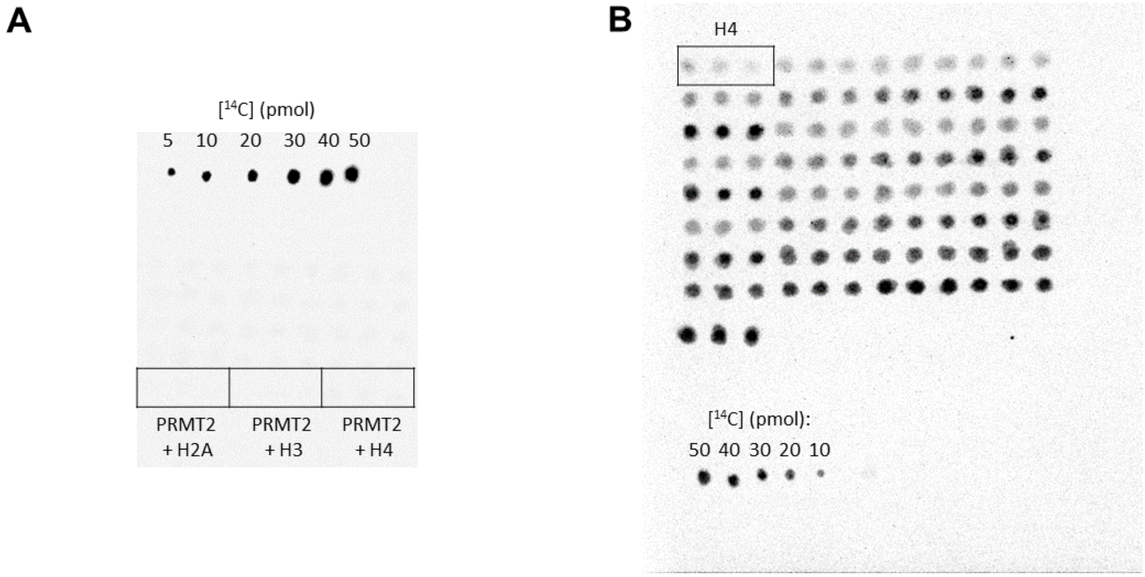

Supporting Figure 20. FBAPS quantitation of individual histone methylation by PRMT2. (A) PRMT2 (10 μM) reaction with 10 μM ^{14}C-SAM and 25 μM of the indicated concentration of recombinant histone for 2 h. (B) The same blot is shown in **Supporting Figure 19** for the purpose of comparing PRMT2-only controls (panel A) to highlight differences in intensities between a PRMT1/2 reaction against H4 and PRMT2 against H4 alone, as well as to quantify absolute levels of C-14 with exposure to a ^{14}C-SAM ladder (both panels). The PRMT2 reaction with histones and ^{14}C-SAM yielded no detectable activity compared to reactions in the presence of PRMT1. Storage phosphor screens were exposed for 72 h before imaging.

```
CLUSTAL multiple sequence alignment by MUSCLE (3.8)
NP_001526.2protein arginine N-methyltransferase 2 isoform 1 [Homo sapiens]
AAI22564.1 protein arginine N-methyltransferase 2 isoform 1 [Mus musculus]


NP_001526.2      MATSGDCPRSESQ-----GEEPAE--CSEAGLLQEG------VQPEEFVAIADYAATDET
AAI22564.1       MEAPGEGPCSESQVIPVLEEDPVDYGC-EMQLLQDGAQLQLQLQPEEFVAIADYTATDET

                  Human SH3 Domain
NP_001526.2      QLSFLRGEKILILRQTTADWWWGERAGCCGYIPANHVGKHVDEYDPEDTWQDEEYFGSYG
AAI22564.1       QLSFLRGEKILILRQTTADWWWGERAGCCGYIPANHLGKQLEEYDPEDTWQDEEYFDSYG

                                    Human Cofactor Binding Domain

NP_001526.2      TLKLHLEMLADQPRTTKYHSVILQNKESLTDKVILDVGCGTGIISLFCAHYARPRAVYAV
AAI22564.1       TLKLHLEMLADQPRTTKYHSVILQNKESLKDKVILDVGCGTGIISLFCAHHARPKAVYAV

NP_001526.2      EASEMAQHTGQLVLQNGFADIITVYQQKVEDVVLPEKVDVLVSEWMGTCLLFEFMIESIL
AAI22564.1       EASDMAQHTSQLVLQNGFADTITVFQQKVEDVVLPEKVDVLVSEWMGTCLLFEFMIESIL

                  Human Beta Barrel         Human  Dimer arm
NP_001526.2      YARDAWLKEDGVIWPTMAALHLVPCSADKDYRSKVLFWDNAYEFNLSALKSLAVKEFFSK
AAI22564.1       YARDTWLKGDGIIWPTTAALHLVPCSAEKDYHSKVLFWDNAYEFNLSALKSLAIKEFFSR

                                       Human Beta Barrel
NP_001526.2      PKYNHILKPEDCLSEPCTILQLDMRTVQISDLETLRGELRFDIRKAGTLHGFTAWFSVHF
AAI22564.1       PKSNHILKPEDCLSEPCTILQLDMRTVQVPDLETMRGELRFDIQKAGTLHGFTAWFSVYF

                                       Human Beta Barrel
NP_001526.2      QSLQEGQPPQVLSTGPFHP------------------------------TTHWKQTLFMM
AAI22564.1       QSLEEGQPQQVLSTGPLHPFLGRTGCQCRGPRWSCQMRPVGDRMLLSCSTTHWKQTLFMM

                                       Human Beta Barrel
NP_001526.2      DDPVPVHTGDVVTGSVVLQRNPVWRRHMSVALSWAVTSRQDPTSQKVGEKVFPIWR
AAI22564.1       DDPVPVHTGDVVTGSVVLQRNPVWRRHMSVSLSWVVTSALDPTSQRVGEKVFPIWR
```

Supporting Figure 21. CLUSTAL multiple PRMT sequence alignment by MUSCLE. PRMT2 from *Homo sapiens,* and *Mus musculus* were aligned and notable human domains were highlighted with structural PRMT feature above (in the same colour). Amino acids in *M. musculus* that do not align and have a different biophysical property compared to the *H. sapiens* sequence were bolded (within the catalytically relevant domains only).

A

```
PRMT2 SH3       33 EEFVAIADYAATDETQLSFLRGEKILILRQTTADWWWGERAGCCGYIPANH 83
                          A+ D+ A +E +L F  GE + +L  +   WW G       G  PAN+
Mona/Gads SH3C 266 RWARALYDFEALEEDELGFRSGEVVEVLDSSNPSWWTGRLHNKLGLFPANY 316
```

B

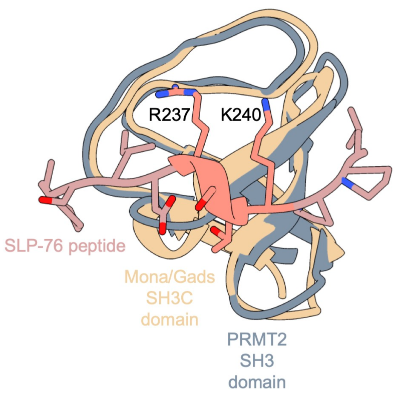

C

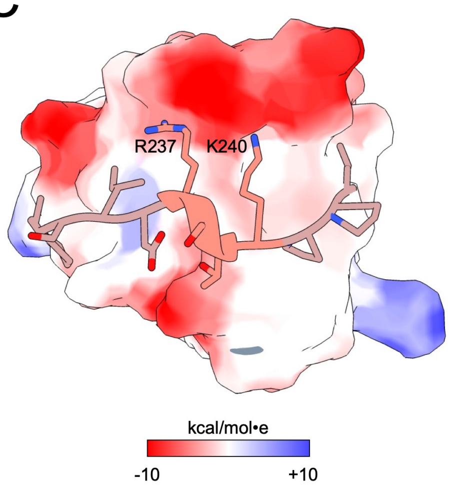

D

SLP-76 231 PAPSID**RSTK**PPL 243

H1.1 1 MSEVALPAPAASTSPEKPSAG**KKAKKPAK**AAAAAKKKPAG 40

H2A.1 1 MSGRGKQGGKARAKAKT**RSSR**AGLQFPVG**RVHRLLR**KGNY 40

H2B.1 1 MPEPAKSAPAPK**KGSK**KAVT**KAQKKDGKKRKRSRK**ESYSV 40

H3.1 1 MARTKQTARKSTGG**KAPR**KQLAT**KAAR**KSAPATGGVKKPH 40

H4 1 MSGRG**KGGK**GLGKGGA**KRHRKVLR**DNIQGITKPAIR**RLAR** 40

Supporting Figure 22. Possible PRMT2 SH3 domain ligands. (A) The human PRMT2 SH3 domain and the Mona/Gads SH3C domain share 31% sequence identity and 47% sequence similarity (+). (B) The Mona/Gads SH3C domain (tan) (PDB 1OEB; amino acids 266-316) in complex with a peptide portion of the T-cell signal transducer SLP-76 (brown) with the R/KXXK motif highlighted (salmon) superimposed with the PRMT2 SH3 domain (PDB 1X2P; amino acids 33-83) showing an r.m.s.d. of 0.975 Å were rendered using Chimera (UCSF). (C) The superimposition described in (B) with the PRMT2 SH3 domain electrostatic surface map is showing basic residues of the RXXK motif interacting with acidic surfaces. (D) The RXXK motif (bolded) found in the SLP-76 sequence can also be found as (R/K)XX(R/K) in bovine histone N-termini, suggesting that the PRMT2 SH3 domain may be able to interact with these sequences. Bovine histone sequences are shown since calf thymus histones are used as methylation substrates in our assays.

Supporting Information Tables

Supporting Table 1. Derived melting temperature (T_m) for PRMT2 from Figure 3. The second derivative of fluorescence with respect to temperature was plotted and the T_m was the x-intercept corresponding to the local maxima observed on the first-derivative plot. R1-R3 are replicates, AVE is average, and SD is standard deviation. Statistical comparisons are shown in **Supporting Table 2**.

5 μM PRMT2 T_{m1} (ºC)			10 μM PRMT2 T_{m1} (ºC)			15 μM PRMT2 T_{m1} (ºC)			PRMT2 5 μM T_{m2} (ºC)		
R1	R2	R3	R1	R2	R3	R1	R2	R3	R1	R2	R3
46.64	46.03	45.27	47.99	47.99	46.96	50.85	49.38	49.72	48.6	48.73	49.1
AVE	SD	N	AVE	SD	N	AVE	SD	N	AVE	SD	N
45.98	0.56	3	47.65	0.49	3	49.98	0.63	3	48.81	0.21	3
**PRMT2 10 μM T_{m2} (ºC)**			**PRMT2 15 μM T_{m2} (ºC)**			**PRMT2 5 μM T_{m1} Storage buffer (2%) (ºC)**			**PRMT2 5 μM T_{m1} Storage buffer (3%) (ºC)**		
R1	R2	R3	R1	R2	R3	R1	R2	R3	R1	R2	R3
52.31	51.1	51.54	53.16	53.16	52.94	48.9	48.99	48.84	53.91	53.82	54.5
AVE	SD	N	AVE	SD	N	AVE	SD	N	AVE	SD	N
51.65	0.5	3	53.09	0.1	3	48.91	0.06	3	54.07	0.3	3

Supporting Table 2. One-way ANOVA statistical testing evaluating the melting temperatures (T_m) from Supporting Figure 3. A statistical level of significance cut-off <0.05 was used.

**Bonferroni's multiple comparisons test**	**ΔT_m (°C)**	**Summary**	**Adjusted P Value**
PRMT2 5 µM T_{m1}vs. PRMT2 10 µM T_{m1}	-1.66	ns	0.055
PRMT2 5 µM T_{m1} vs. PRMT2 15 µM T_{m1}	-4.00	***	0.00022
PRMT2 5 µM T_{m1} vs. PRMT2 5 µM + 2% glycerol T_{m1}	-2.93	**	0.0019
PRMT2 10 µM T_{m1} vs. PRMT2 15 µM T_{m1}	-2.34	**	0.0082
PRMT2 10 µM T_{m1} vs. PRMT2 5 µM + 2% glcyerol T_{m1}	-1.27	ns	0.19
PRMT2 5 µM T_{m2} vs. PRMT2 10 µM T_{m2}	-2.84	****	0.000015
PRMT2 5 µM T_{m2} vs. PRMT2 15 µM T_{m2}	-4.28	****	0.00000032
PRMT2 5 µM T_{m2} vs. PRMT2 5 µM + 2% glycerol T_{m2}	-0.10	ns	>0.99
PRMT2 5 µM T_{m2} vs. PRMT2 5 µM + 3% glycerol T_{m2}	-5.26	****	0.000000043
PRMT2 10 µM T_{m2} vs. PRMT2 15 µM T_{m2}	-1.44	**	0.0046
PRMT2 10 µM T_{m2} vs. 2% glycerol T_{m2}	2.74	****	0.000021
PRMT2 15 µM T_{m2} vs. 3% glycerol T_{m2}	-0.98	ns	0.059

Supporting Table 3. Derived melting temperature for PRMT2 (T_{m1}) with ligands from Figure 1. The second derivative of fluorescence with respect to temperature was plotted and the T_m was the x-intercept which corresponded with the local maxima observed on the first-derivative plot. Starred values (*) were excluded due to experimental issues.

[SAH] (µM)	T_{m1}			T_{m2}			[H2A] (µM)	T_{m1} (°C)		
	R1	R2	R3	R1	R2	R3		R1	R2	R3
0	46.63	46.16	46.75	49.72	49.17	50.11	0	52.91	52.51	52.55
	Ave	SD	N	Ave	SD	N		Ave	SD	N
	46.51	0.25	3	49.67	0.39	3		52.66	0.18	3
	R1	R2	R3	R1	R2	R3		R1	R2	R3
1	46.71	47.07*	47.16	50.43	53.03*	51.03	1	53.35	52.68	52.59
	Ave	SD	N	Ave	SD	N		Ave	SD	N
	46.93	0.23	2	50.73	0.3	2		52.87	0.34	3
	R1	R2	R3	R1	R2	R3		R1	R2	R3
5	46.57	47.08	46.2	49.94	50.92	49.9	5	52.63	52.64	52.80
	Ave	SD	N	Ave	SD	N		Ave	SD	N
	46.62	0.36	3	50.25	0.47	3		52.69	0.08	3
	R1	R2	R3	R1	R2	R3		R1	R2	R3
10	47	46.87	47.17	50.47	50.17	50.71	10	52.91	52.92	52.85
	Ave	SD	N	Ave	SD	N		Ave	SD	N
	47.01	0.12	3	50.45	0.22	3		52.89	0.03	3
	R1	R2	R3	R1	R2	R3		R1	R2	R3
50	46.79	46.76	46.39	50.27	50.41	49.48	50	53.20	53.29	53.27
	Ave	SD	N	Ave	SD	N		Ave	SD	N
	46.65	0.18	3	50.05	0.41	3		53.25	0.04	3
	R1	R2	R3	R1	R2	R3		R1	R2	R3
100	46.93	46.73	46.84	50.5	50.32	50.01	100	53.79	53.81	53.76
	Ave	SD	N	Ave	SD	N		Ave	SD	N
	46.83	0.08	3	50.28	0.2	3		53.79	0.02	3
	R1	R2	R3	R1	R2	R3		R1	R2	R3
500	46.61	46.46	46.06	49.9	50.29	49.28	500	54.66	54.66	54.55
	Ave	SD	N	Ave	SD	N		Ave	SD	N
	46.38	0.23	3	49.82	0.41	3		54.62	0.05	3

[H3] (μM)	T_{m1}			[H4] (μM)	T_{m1}		
0	R1	R2	R3	0	R1	R2	R3
	47.17	46.54	47.24		46.53	45.89	45.71
	Ave	SD	N		Ave	SD	N
	46.98	0.31	3		46.04	0.35	3
1	R1	R2	R3	1	R1	R2	R3
	47.1		47.62		46.41	46.49	46.55
	Ave	SD	N		Ave	SD	N
	47.36	0.26	3		46.48	0.06	3
5	R1	R2	R3	5	R1	R2	R3
	47.06	46.9	47.25		46.43	46.51	46.33
	Ave	SD	N		Ave	SD	N
	47.07	0.14	3		46.42	0.07	3
10	R1	R2	R3	10	R1	R2	R3
	47.49	47.33	47.39		46.78	46.17	46.31
	Ave	SD	N		Ave	SD	N
	47.4	0.06	3		46.42	0.26	3
50	R1	R2	R3	50	R1	R2	R3
	51.59	51.29	51.49		47.15	47.26	46.31
	Ave	SD	N		Ave	SD	N
	51.46	0.13	3		46.9	0.42	3
100	R1	R2	R3	100	R1	R2	R3
	52.03	52.27	52.13		47.09	47.57	47.46
	Ave	SD	N		Ave	SD	N
	52.14	0.1	3		47.37	0.2	3
500	R1	R2	R3	500	R1	R2	R3
	54.71	54.76	54.77		52.18	51.71	51.86
	Ave	SD	N		Ave	SD	N
	54.75	0.03	3		51.92	0.2	3

Supporting Table 4. One-way ANOVA statistical testing evaluating the melting temperatures (T_{m1}) from Figure 1. A statistical level of significance cut-off <0.05 was used.

	Comp.	ΔT_m (°C)	Sum.	P Value
**SAH T_{m1}**	0 vs. 1	-0.42	ns	0.7
	0 vs. 5	-0.10	ns	>0.99
	0 vs. 10	-0.50	ns	0.23
	0 vs. 50	-0.13	ns	>0.99
	0 vs. 100	-0.32	ns	>0.99
	0 vs. 500	0.140	ns	>0.99

	Comp.	ΔT_m (°C)	Sum	Adjusted P Value
**H2A T_{m1}**	0 vs. 1	0.22	ns	0.59
	0 vs. 5	0.03	ns	>0.99
	0 vs. 10	0.24	ns	0.46
	0 vs. 50	0.6	**	1.57×10^{-3}
	0 vs. 100	1.13	****	1.58×10^{-6}
	0 vs. 500	1.97	****	1.33×10^{-9}

	Comp.	ΔT_m (°C)	Sum.	P Value
**H3 T_{m1}**	0 vs. 1	0.38	ns	>0.99
	0 vs. 5	0.09	ns	>0.99
	0 vs. 10	0.42	ns	>0.99
	0 vs. 50	4.47	****	8.35×10^{-12}
	0 vs. 100	5.16	****	1.02×10^{-12}
	0 vs. 500	7.77	****	2.00×10^{-15}

	Comp.	ΔT_m (°C)	Sum.	P Value
**SAH T_{m2}**	0 vs. 1	-1.06	ns	0.13
	0 vs. 5	-0.58	ns	0.87
	0 vs. 10	-0.78	ns	0.32
	0 vs. 50	-0.39	ns	>0.99
	0 vs. 100	-0.61	ns	0.77
	0 vs. 500	-0.16	ns	>0.99

	Comp.	ΔT_m (°C)	Sum.	P Value
**H4 T_{m1}**	0 vs. 1	-0.44	ns	0.74
	0 vs. 5	-0.38	ns	>0.99
	0 vs. 10	-0.38	ns	>0.99
	0 vs. 50	-0.86	*	0.03
	0 vs. 100	-1.33	***	6.4×10^{-4}
	0 vs. 500	-5.87	****	8.61×10^{-13}

Supporting Table 5. Derived melting temperatures for PRMT2 (T_{m1}) from Supporting Figure 6. The second derivative of fluorescence with respect to temperature was plotted and the T_m was the x-intercept which corresponded with the local maxima observed on the first-derivative plot.

T_m PRMT2 (ºC)											
**PRMT2 (apo)**			**SAH**			**H3**			**SAH + H3**		
**R1**	**R1**	**R2**	**R1**	**R1**	**R1**	**R1**	**R2**	**R3**	**R1**	**R2**	**R3**
47.17	46.54	47.24	46.50	46.78	46.41	54.71	54.76	54.77	53.89	54.01	53.89
**AVE**	**SD**	**N**	**AVE**	**SD**	**N**	**AVE**	**SD**	**N**	**AVE**	**SD**	**N**
46.98	0.31	3	46.56	0.15	3	54.75	0.03	3	53.93	0.06	3

Supporting Table 6. One-way ANOVA statistical testing evaluating the melting temperatures (T_{m1}) from Supporting Figure 6. A statistical level of significance cut-off <0.05 was used.

Bonferroni's multiple comparisons test	ΔT_m (°C)	Summary	Adjusted P Value
PRMT2 vs. PRMT2 + SAH	0.42	ns	>0.99
PRMT2 vs. PRMT2 + H3	-7.77	****	$<1.00 \times 10^{-15}$
PRMT2 vs. PRMT2 + SAH + H3	-6.95	****	$<1.00 \times 10^{-15}$
PRMT2 + SAH vs. PRMT2 + H3	-8.19	****	$<1.00 \times 10^{-15}$
PRMT2 + SAH vs. PRMT2 + SAH + H3	-7.37	****	$<1.00 \times 10^{-15}$
PRMT2 + H3 vs. PRMT2 + SAH + H3	0.82	*	0.02

Supporting Table 7. Derived melting temperature for PRMT2H112Q (T_{m1}) from Supporting Figure 7. The second derivative of fluorescence with respect to temperature was plotted and the T_m was the x-intercept which corresponded with the local maxima observed on the first-derivative plot.

PRMT2H112Q			PRMT2H112Q + SAH			PRMT2H112Q + H3			PRMT2H112Q + H3 + SAH		
R1	R2	R3	R1	R2	R3	R1	R2	R3	R1	R2	R3
43.37	43.47	43.92	44.41	43.52	43.56	43.46	42.94	44.10	43.04	42.96	44.03
Ave	SD	N	Ave	SD	N	Ave	SD	N	Ave	SD	N
43.58	0.24	3	43.83	0.41	3	43.50	0.47	3	43.34	0.49	3

Supporting Table 8. One-way ANOVA statistical testing evaluating the melting temperatures (T_{m1}) from Supporting Figure 7. A statistical level of significance cut-off <0.05 was used.

Bonferroni's multiple comparisons test	ΔT_m (°C)	Summary	Adjusted P Value
PRMT2H112Q vs. PRMT2H112Q + SAH	-0.25	ns	>0.99
PRMT2H112Q vs. PRMT2H112Q + H3	0.084	ns	>0.99
PRMT2H112Q vs. PRMT2H112Q + H3 + SAH	0.24	ns	>0.99
PRMT2H112Q + SAH vs. PRMT2H112Q + H3	0.33	ns	>0.99
PRMT2H112Q + SAH vs. PRMT2H112Q + H3 + SAH	0.49	ns	>0.99
PRMT2H112Q + H3 vs. PRMT2H112Q + H3 + SAH	0.16	ns	>0.99

Supporting Table 9. Derived melting temperature for PRMT2ΔSH3 (T_{m1}) from Supporting Figure 8. The second derivative of fluorescence with respect to temperature was plotted and the T_m was the x-intercept which corresponded with the local maxima observed on the first-derivative plot.

T_m (ºC)											
PRMT2ΔSH3			PRMT2ΔSH3 + H3 Peptide			PRMT2ΔSH3 + SAH			PRMT2ΔSH3 + H3 Peptide + SAH		
R1	R2	R3	R1	R2	R3	R1	R2	R3	R1	R2	R3
40.09	39.16	39.34	41.36	42.34	42.81	40.34	40.47	39.96	41.74	42.28	43.77
AVE	SD	N	AVE	SD	N	AVE	SD	N	AVE	SD	N
39.53	0.40	3.00	42.17	0.60	3.00	40.26	0.22	3.00	42.60	0.86	3.00

Supporting Table 10. One-way ANOVA statistical testing evaluating the for PRMT2ΔSH3 melting temperatures (T_{m1}) from Supporting Figure 8. A statistical level of significance cut-off <0.05 was used.

PRMT2ΔSH3			
Bonferroni's multiple comparisons test	ΔT_m (°C)	Summary	Adjusted P Value
PRMT2ΔSH3 vs. PRMT2ΔSH3 + H3 Peptide	-2.64	**	0.0029
PRMT2ΔSH3 vs. PRMT2ΔSH3 + SAH	-0.73	ns	0.94
PRMT2ΔSH3 vs. PRMT2ΔSH3 + H3 Peptide + SAH	-3.07	**	0.001
PRMT2ΔSH3 + H3 Peptide vs. PRMT2ΔSH3 + SAH	1.91	*	0.021
PRMT2ΔSH3 + H3 Peptide vs. PRMT2ΔSH3 + H3 Peptide + SAH	-0.43	ns	>0.99
PRMT2ΔSH3 + SAH vs. PRMT2ΔSH3 + H3 Peptide + SAH	-2.34	**	0.0062

Supporting Table 11. Derived melting temperatures for PRMT2ΔSH3 (T_m) with H3 peptide from Supporting Figure 8. The second derivative of fluorescence with respect to temperature was plotted and the T_m was the x-intercept that corresponded with local maxima observed on the first-derivative plot.

[H3 peptide] (uM)	T_m PRMT2ΔSH3		
	Ave	SD	N
0	44.49	0.35	3
5	44.33	0.24	3
10	44.93	0.46	3
50	44.57	0.15	3
100	44.80	0.40	3
500	46.64	0.48	3

Supporting Table 12. Derived melting temperature for PRMT1 (T_{m1}) from Supporting Figure 9. The second derivative of fluorescence with respect to temperature was plotted and the T_m was the x-intercept which corresponded with local maxima observed on the first-derivative plot.

T_m PRMT1 (ºC)											
Apo			H3			SAH			H3 + SAH		
Ave	SD	N	Ave	SD	N	Ave	SD	N	Ave	SD	N
49.34	0.17	3	50.61	0.21	3	53.28	0.12	3	59.84	0.04	3

Supporting Table 13. Derived melting temperature for PRMT1 (T_{m1}) from Supporting Figure 9. The second derivative of fluorescence with respect to temperature was plotted and the T_m was the x-intercept which corresponded with local maxima observed on the first-derivative plot.

[H3 Peptide] (µM)	T_m PRMT1		
0	R1	R2	R3
	54.19	54.42	54.44
	Ave	SD	N
	54.35	0.11	3
0.5	R1	R2	R3
	54.52	54.45	54.58
	Ave	SD	N
	54.51	0.05	3
1	R1	R2	R3
	54.59	54.47	54.3
	Ave	SD	N
	54.45	0.12	3
5	R1	R2	R3
	54.48	54.85	53.92
	Ave	SD	N
	54.42	0.38	3
10	R1	R2	R3
	54.46	54.85	54.59
	Ave	SD	N
	54.63	0.16	3
50	R1	R2	R3
	55.51	55.87	55.68
	Ave	SD	N
	55.69	0.14	3
100	R1	R2	R3
	56.18	56.1	56.43
	Ave	SD	N
	56.24	0.14	3
500	R1	R2	R3
	58.12	58.32	58.38
	Ave	SD	N
	58.28	0.11	3

Supporting Table 14. One-way ANOVA statistical testing evaluating the melting temperatures (T_{m1}) from Supporting Figure 7. A statistical level of significance cut-off <0.05 was used.

Bonferroni's multiple comparisons test	ΔT_m (°C)	Summary	Adjusted P Value
apo vs. H3	-1.28	****	0.000017
apo vs. SAH	-3.95	****	0.0000000025
apo vs. H3 + SAH	-10.50	****	0.0000000000010
**Bonferroni's multiple comparisons test**	**ΔT_m (°C)**	**Summary**	**Adjusted P Value**
0 vs. 0.5	-0.16	ns	>0.99
0 vs. 1	-0.0042	ns	>0.99
0 vs. 5	0.24	ns	>0.99
0 vs. 10	-0.090	ns	>0.99
0 vs. 50	-1.2	*	0.042
0 vs. 100	-1.8	***	0.00099
0 vs. 500	-3.8	****	0.000000065

Supporting Table 15. Derived melting temperature for PRMT1/2 complex (T_{m1}/T_{m2}) from Figure 2. The second derivative of fluorescence with respect to temperature was plotted and the T_m was the x-intercept which corresponded with the local maxima observed on the first-derivative plot.

**apo**								
5 µM PRMT2 T_{m1}			5 µM PRMT1			5 µM PRMT2 + 5 µM PRMT1		
AVE	SD	N	AVE	SD	N	AVE	SD	N
45.98	0.56	3	49.34	0.17	3	49.16	0.08	3

**SAH**								
5 µM PRMT2 +			5 µM PRMT1			5 µM PRMT2 + 5 µM PRMT1		
AVE	SD	N	AVE	SD	N	AVE	SD	N
46.14	0.35	3	53.28	0.12	3	53.52	0.07	3

**H3 peptide**											
5 µM PRMT2			5 µM PRMT1			5 µM PRMT2 + 5 µM PRMT1 T_{m1}			5 µM PRMT2 + 5 µM PRMT1 T_{m2}		
AVE	SD	N	AVE	SD	N	AVE	SD	N	AVE	SD	N
53.31	0.07	3	50.61	0.21	3	53.58	0.11	3	57.74	0.23	3

**H3 peptide + SAH**								
5 µM PRMT2 + H3 + SAH			5 µM PRMT1 + H3 + SAH			5 µM PRMT2 + 5 µM PRMT1 + H3 + SAH		
AVE	SD	N	AVE	SD	N	AVE	SD	N
55.11	0.02	3	59.83	0.04	3	58.31	0.12	3

Supporting Table 16. One-way ANOVA statistical comparison of the melting temperatures obtained for PRMT1 and PRMT2 (from Figure 2). A statistical level of significance cut-off <0.05 was used.

**Bonferroni's multiple comparisons test**	**T_{m1}**	**T_{m2}**	**ΔT_m (°C)**	**P-value**	**Significance**
**apo**					
PRMT2 vs. PRMT1/ 2	■		3.18	6.51×10^{-10}	****
PRMT1 vs. PRMT1/2	■		-0.18	>0.99	ns
**SAH**					
PRMT2 vs. PRMT1/2	■		7.38	$<1.00 \times 10^{-15}$	****
PRMT1 vs. PRMT1/2	■		0.24	>0.99	ns
**H3**					
PRMT2 vs. PRMT1/2	■		3.23	2.13×10^{-11}	****
PRMT1 vs. PRMT1/:2	■		0.53	0.489	ns
PRMT2 vs. PRMT1/2		■	-0.94	0.00364	**
PRMT1 vs. PRMT1/2		■	3.63	2.51×10^{-12}	****
**H3 + SAH**					
PRMT2 vs. PRMT1/2	■		3.2	1.09×10^{-14}	****
PRMT1 vs. PRMT1/2	■		-1.53	1.76×10^{-6}	****

Supporting Table 17. Derived melting temperature for PRMT1 and PRMT2ΔSH3 from Figure 2. The second derivative of fluorescence with respect to temperature was plotted and the T_m was the x-intercept which corresponded with the local maxima on the first-derivative plot.

**apo**											
**PRMT2ΔSH3**			**PRMT2ΔSH3 + PRMT1**			**PRMT1**					
R1	R2	R3	R1	R2	R3	R1	R2	R3			
40.09	39.16	39.34	**45.59***	47.42	47.98	49.56	49.15	49.3			
AVE	SD	N	AVE	SD	N	AVE	SD	N			
39.53	0.4	3	47.7	0.28	2	49.34	0.17	3			
**SAH**											
**PRMT2ΔSH3**			**PRMT2ΔSH3 + PRMT1 T_{m1}**			**PRMT1**			**PRMT2ΔSH3 + PRMT1 T_{m2}**		
R1	R2	R3	R1	R2	R3	R1	R2	R3	R1	R2	R3
40.34	40.47	39.96	53.31	53.61	53.57	53.12	53.32	53.41	63.93	64.09	63.12
AVE	SD	N	AVE	SD	N	AVE	SD	N	AVE	SD	N
40.26	0.22	3	53.5	0.13	3	53.28	0.12	3	63.71	0.43	3
**H3 peptide**											
**PRMT2ΔSH3**			**PRMT2ΔSH3 + PRMT1 T_{m1}**			**PRMT1**			**PRMT2ΔSH3 + PRMT1 T_{m2}**		
R1	R2	R3	R1	R2	R3	R1	R2	R3	R1	R2	R3
41.36	42.34	42.81	49.29	49.42	49.32	50.35	50.62	50.86	57.32	57.32	57.4
AVE	SD	N	AVE	SD	N	AVE	SD	N	AVE	SD	N
42.17	0.6	3	49.34	0.06	3	50.61	0.21	3	57.35	0.04	3
**H3 peptide + SAH**											
**PRMT2ΔSH3**			**PRMT2ΔSH3 + PRMT1**			**PRMT1**					
R1	R2	R3	R1	R2	R3	R1	R2	R3			
41.74	42.28	43.77	57.37	57.36	57.37	59.89	59.8	59.81			
AVE	SD	N	AVE	SD	N	AVE	SD	N			
42.6	0.86	3	57.37	0.01	3	59.83	0.04	3			

Supporting Table 18. One-way ANOVA statistical comparison of the melting temperatures obtained for PRMT1 and PRMT2ΔSH3 (from Figure 2. A statistical level of significance cut-off <0.05 was used.

Bonferroni's multiple comparisons test	ΔT_m (°C)	Summary	Adjusted P Value
**apo**			
PRMT2ΔSH3 vs. PRMT1/2ΔSH3	8.17	****	5.90×10^{-8}
PRMT1/2ΔSH3 vs. PRMT1	1.64	**	0.002722
**SAH**			
PRMT2ΔSH3 vs. PRMT1/2ΔSH3 T_{m1}	13.24	****	2.32×10^{-13}
PRMT2ΔSH3 vs. PRMT1/2ΔSH3 T_{m2}	23.45	****	$<1.0 \times 10^{-15}$
PRMT1/2ΔSH3 T_{m1} vs. PRMT1	-0.22	ns	>0.99
PRMT1/2ΔSH3 T_{m2} vs. PRMT1	-10.43	****	2.50×10^{-12}
**H3 peptide**			
PRMT2ΔSH3 vs. PRMT1/2ΔSH3 T_{m1}	7.17	****	7.97×10^{-10}
PRMT2ΔSH3 vs. PRMT1/2ΔSH3 T_{m2}	15.18	****	4.61×10^{-13}
PRMT1/2ΔSH3 T_{m1} vs. PRMT1	1.27	**	0.00577
PRMT1/2ΔSH3 T_{m2} vs. PRMT1	-6.74	****	1.47×10^{-9}
**H3 peptide + SAH**			
PRMT2ΔSH3 vs. PRMT1/2ΔSH3	14.77	****	1.65×10^{-6}
PRMT1/2ΔSH3 vs. PRMT1	-2.46	**	0.001456

Supporting Table 19. Derived melting temperatures for PRMT1 and PRMT2H112Q from Figure 2. The second derivative of fluorescence with respect to temperature was plotted and the T_m was the x-intercept which corresponded with the local maxima on the first-derivative plot.

apo											
PRMT2H112Q			PRMT1			PRMT1/2H112Q T_{m1}			PRMT1/2H112Q T_{m2}		
R1	R2	R3	R1	R2	R3	R1	R2	R3	R1	R2	R3
43.37	43.47	43.92	49.39	49.33	49.27	42.32	42.09	41.50	50.13	50.23	50.33
AVE	SD	N	AVE	SD	N	AVE	SD	N	AVE	SD	N
43.58	0.24	3	49.33	0.05	3	41.97	0.35	3	50.23	0.08	3
SAH											
PRMT2H112Q			PRMT1			PRMT1/2H112Q Tm1			PRMT1/2H112Q T_{m2}		
R1	R2	R3	R1	R2	R3	R1	R2	R3	R1	R2	R3
44.41	43.52	43.56	58.02	57.59	57.84	44.58	43.60	44.14	57.83	57.19	57.64
AVE	SD	N	AVE	SD	N	AVE	SD	N	AVE	SD	N
43.83	0.41	3	57.82	0.18	3	44.11	0.40	3	57.56	0.27	3
H3 peptide											
PRMT2H112Q			PRMT1			PRMT1/2H112Q T_{m1}			PRMT1/2H112Q T_{m2}		
R1	R2	R3	R1	R2	R3	R1	R2	R3	R1	R2	R3
43.46	42.94	44.10	50.33	50.39	50.35	42.62	42.72	43.16	50.75	50.72	50.88
AVE	SD	N	AVE	SD	N	AVE	SD	N	AVE	SD	N
43.50	0.47	3	50.36	0.02	3	42.83	0.24	3	50.78	0.07	3
SAH + H3 peptide											
PRMT2H112Q			PRMT1			PRMT1/2H112Q T_{m1}			PRMT1/2H112Q T_{m2}		
R1	R2	R3	R1	R2	R3	R1	R2	R3	R1	R2	R3
43.04	42.96	44.03	58.77	59.12	59.19	45.38	45.06	44.63	57.51	57.20	57.73
AVE	SD	N	AVE	SD	N	AVE	SD	N	AVE	SD	N
43.34	0.49	3	59.02	0.18	3	45.02	0.31	3	57.48	0.22	3

Supporting Table 20. One-way ANOVA statistical comparison of the melting temperatures obtained for PRMT1 and PRMT2H112Q (from Figure 2). A statistical level of significance cut-off <0.05 was used.

Bonferroni's multiple comparisons test	ΔT_m (°C)	Summary	Adjusted P Value
**apo**			
PRMT2H112Q vs. PRMT1/2H112Q Tm_1	-1.61	**	0.00211
PRMT1 vs. PRMT1/2H112Q Tm_2	0.90	****	0.000086
**SAH**			
**Bonferroni's multiple comparisons test**	**Mean Diff.**	**Summary**	**Adjusted P Value**
PRMT2H112Q vs. PRMT1/2H112Q Tm_1	0.28	ns	>0.99
PRMT1 vs. PRMT1/2H112Q Tm_2	-0.26	ns	>0.99
**H3 peptide**			
**Bonferroni's multiple comparisons test**	**Mean Diff.**	**Summary**	**Adjusted P Value**
PRMT2H112Q vs. PRMT1/2H112Q Tm_1	-0.67	ns	>0.99
PRMT1 vs. PRMT1/2H112Q Tm_2	0.42	ns	0.13
**H3 peptide + SAH**			
**Bonferroni's multiple comparisons test**	**Mean Diff.**	**Summary**	**Adjusted P Value**
PRMT2H112Q vs. PRMT1/2H112Q Tm_1	1.68	**	0.00139
PRMT1 vs. PRMT1/2H112Q Tm_2	-1.54	****	0.0000000585

Supporting Table 21. Derived melting temperature for PRMT1 and PRMT2 (at different concentrations) from Supporting Figure 13. The second derivative of fluorescence with respect to temperature was plotted and the T_m was the x-intercept which corresponded with the local maxima observed on the first-derivative plot.

	**0 µM PRMT1**						**1 µM PRMT1**						**5 µM PRMT1**						**10 µM PRMT1**					
**0 µM PRMT2**	T_{m1}			T_{m2}			T_{m1}			T_{m2}			T_{m1}			T_{m2}			T_{m1}			T_{m2}		
							48.25	48.42	48.3				49.56	49.15	49.3				50.14	50.05	50.09			
	Ave	SD	N	Ave	SD	N	Ave	SD	N	Ave	SD	N	Ave	SD	N	Ave	SD	N	Ave	SD	N	Ave	SD	N
							48.32	0.07	3				49.34	0.17	3				50.1	0.03	3			
**1 µM PRMT2**	T_{m1}			T_{m2}			T_{m1}			T_{m2}			T_{m1}			T_{m2}			T_{m1}			T_{m2}		
	43.22	43.39	42.36	46.2	46.21	45.83	48.31	48.42	48.42	52.67	52.19	53.58	49.34	49.14	49.41				49.87	50.1	50.19			
	Ave	SD	N	Ave	SD	N	Ave	SD	N	Ave	SD	N	Ave	SD	N	Ave	SD	N	Ave	SD	N	Ave	SD	N
	42.99	0.45	3	46.08	0.17	3	48.38	0.05	3	52.81	0.58	3	49.3	0.11	3				50.05	0.13	4			
**5 µM PRMT2**	T_{m1}			T_{m2}			T_{m1}			T_{m2}			T_{m1}			T_{m2}			T_{m1}			T_{m2}		
	46.64	46.03	45.27	48.6	48.73	49.1	47.32	47.44	47.01	51.59	52.89	52.94	49.23	49.05	49.2				49.81	49.95	49.73			
	Ave	SD	N	Ave	SD	N	Ave	SD	N	Ave	SD	N	Ave	SD	N	Ave	SD	N	Ave	SD	N	Ave	SD	N
	45.98	0.56	3	48.81	0.21	3	47.26	0.18	3	52.47	0.63	3	49.16	0.08	3				49.83	0.09	5			
**10 µM PRMT2**	T_{m1}			T_{m2}			T_{m1}			T_{m2}			T_{m1}			T_{m2}			T_{m1}			T_{m2}		
	48.89	48.7	49.16	52.3	51.78	51.87	47.25	46.77	46.28	54.74	54.94	55.06	48.8	48.79	49.06	52.91	53.52	53.27	49.75	50.21	50.06	54.5	54.72	54.24
	Ave	SD	N	Ave	SD	N	Ave	SD	N	Ave	SD	N	Ave	SD	N	Ave	SD	N	Ave	SD	N	Ave	SD	N
	48.92	0.19	3	51.99	0.22	3	46.77	0.39	3	54.91	0.13	3	48.88	0.12	3	53.24	0.25	3	50	0.19	6	54.49	0.19	6

SAH	0 µM PRMT1						1 µM PRMT1						5 µM PRMT1						10 µM PRMT1					
0 µM PRMT2	T_{m1}			T_{m2}			T_{m1}			T_{m2}			T_{m1}			T_{m2}			T_{m1}			T_{m2}		
							52.25	52.47	52.53				53.12	53.32	53.41				54.22	54.16	53.92			
	Ave	SD	N	Ave	SD	N	Ave	SD	N	Ave	SD	N	Ave	SD	N	Ave	SD	N	Ave	SD	N	Ave	SD	N
							52.42	0.12	3				53.28	0.12	3				54.1	0.13	3			
1 µM PRMT2	T_{m1}			T_{m2}			T_{m1}			T_{m2}			T_{m1}			T_{m2}			T_{m1}			T_{m2}		
	42.7	41.64	41.48	46.02	46.36	45.72	48.18	47.92	48.21	52.53	52.16	52.58	48.73	48.92	47.99	53.31	53.59	53.22	49.84		49.83	54.27	54.25	54.57
	Ave	SD	N	Ave	SD	N	Ave	SD	N	Ave	SD	N	Ave	SD	N	Ave	SD	N	Ave	SD	N	Ave	SD	N
	41.94	0.54	3	46.03	0.26	3	48.1	0.13	3	52.42	0.19	3	48.55	0.4	3	53.37	0.16	3	49.83	0	2	54.36	0.15	3
5 µM PRMT2	T_{m1}			T_{m2}			T_{m1}			T_{m2}			T_{m1}			T_{m2}			T_{m1}			T_{m2}		
	46.61	45.78	46.02	49.66	50.07		48.94	48.45		53.04	52.64	52.57	48.35	48.16	48.32	53.43	53.61	53.54	49.61	49.41		54.23	54.19	53.82
	Ave	SD	N	Ave	SD	N	Ave	SD	N	Ave	SD	N	Ave	SD	N	Ave	SD	N	Ave	SD	N	Ave	SD	N
	46.14	0.35	3	49.86	0.2	2	48.69	0.24	2	52.75	0.21	3	48.28	0.08	3	53.52	0.07	3	49.51	0.1	2	54.08	0.18	3
10 µM PRMT2	T_{m1}			T_{m2}			T_{m1}			T_{m2}			T_{m1}			T_{m2}			T_{m1}			T_{m2}		
	49.12	48.73	48.75	51.77	51.78	51.66	54.22	54.12	54.16				53.4	53.5	53				50.3	50.91		54.57	54.43	54.33
	Ave	SD	N	Ave	SD	N	Ave	SD	N	Ave	SD	N	Ave	SD	N	Ave	SD	N	Ave	SD	N	Ave	SD	N
	48.87	0.18	3	51.74	0.06	3	54.17	0.04	3				53.3	0.22	3				50.6	0.3	2	54.45	0.1	3

H3	0 µM PRMT1						1 µM PRMT1						5 µM PRMT1						10 µM PRMT1					
**0 µM PRMT2**	T_{m1}			T_{m2}			T_{m1}			T_{m2}			T_{m1}			T_{m2}			T_{m1}			T_{m2}		
							44.52	44.64	44.41				53.86	54.15	54.37				55.39	55.46	55.31			
	Ave	SD	N	Ave	SD	N	Ave	SD	N	Ave	SD	N	Ave	SD	N	Ave	SD	N	Ave	SD	N	Ave	SD	N
							44.52	0.09	3				54.12	0.21	3				55.39	0.06	3			
**1 µM PRMT2**	T_{m1}			T_{m2}			T_{m1}			T_{m2}			T_{m1}			T_{m2}			T_{m1}			T_{m2}		
	57.2	57.13	57.13				57.07	56.92	57.15	59.49	58.93		53.91	54.13	53.97	57.58	57.63	58.04	55.32	55.36	55.12	58.94	58.98	58.37
	Ave	SD	N	Ave	SD	N	Ave	SD	N	Ave	SD	N	Ave	SD	N	Ave	SD	N	Ave	SD	N	Ave	SD	N
	57.15	0.03	3				57.05	0.09	3	59.21	0.28	2	54	0.09	3	57.75	0.21	3	55.27	0.1	3	58.76	0.28	3
**5 µM PRMT2**	T_{m1}			T_{m2}			T_{m1}			T_{m2}			T_{m1}			T_{m2}			T_{m1}			T_{m2}		
	56.74	56.9	56.79				54	54.02		58.87	57.5	57.61	53.47	53.73	53.55	58.02	57.74	57.45	54.46	54.74	54.64	57.91	58.86	58.11
	Ave	SD	N	Ave	SD	N	Ave	SD	N	Ave	SD	N	Ave	SD	N	Ave	SD	N	Ave	SD	N	Ave	SD	N
	56.81	0.07	3				54.01	0.01	3	57.99	0.62	2	53.58	0.11	3	57.74	0.23	3	54.61	0.12	3	58.29	0.41	3
**10 µM PRMT2**	T_{m1}			T_{m2}			T_{m1}			T_{m2}			T_{m1}			T_{m2}			T_{m1}			T_{m2}		
	57.05	57.27	57.11				58.89	58.45	58.56				53.31	53.06	52.84	57.51	57.79	57.7	54.11	54.36	54	58.97	58.93	58.59
	Ave	SD	N	Ave	SD	N	Ave	SD	N	Ave	SD	N	Ave	SD	N	Ave	SD	N	Ave	SD	N	Ave	SD	N
	57.14	0.09	3				58.63	0.19	3				53.07	0.19	3	57.66	0.12	3	54.16	0.15	3	58.83	0.17	3

Supporting Information

H3 / SAH	0 µM PRMT1						1 µM PRMT1						5 µM PRMT1						10 µM PRMT1					
0 µM PRMT2	T_{m1}			T_{m2}			T_{m1}			T_{m2}			T_{m1}			T_{m2}			T_{m1}			T_{m2}		
							55.45	54.59	55.58				59.89	59.8	59.81	64.05	63.88	63.54	59.75	59.88	59.73	67.38	67.12	66.67
	Ave	SD	N	Ave	SD	N	Ave	SD	N	Ave	SD	N	Ave	SD	N	Ave	SD	N	Ave	SD	N	Ave	SD	N
							55.21	0.44	3				59.83	0.04	3	63.82	0.21	3	59.79	0.07	3	67.06	0.29	3
1 µM PRMT2	T_{m1}			T_{m2}			T_{m1}			T_{m2}			T_{m1}			T_{m2}			T_{m1}			T_{m2}		
	55.23	55.36	55.23				55.15	55.41	55.15				59.22	59.28	59.11	67.93	68.43	67.96	59.5	59.82	59.57	68.69	68.61	68.95
	Ave	SD	N	Ave	SD	N	Ave	SD	N	Ave	SD	N	Ave	SD	N	Ave	SD	N	Ave	SD	N	Ave	SD	N
	55.27	0.06	3				55.23	0.12	3				59.2	0.07	3	68.11	0.23	3	59.63	0.14	3	68.75	0.14	3
5 µM PRMT2	T_{m1}			T_{m2}			T_{m1}			T_{m2}			T_{m1}			T_{m2}			T_{m1}			T_{m2}		
	55.1	55.1	55.13				56.27	55.98	56.03				58.33	58.44	58.15				58.87	58.65	58.65			
	Ave	SD	N	Ave	SD	N	Ave	SD	N	Ave	SD	N	Ave	SD	N	Ave	SD	N	Ave	SD	N	Ave	SD	N
	55.11	0.02	3				56.09	0.12	3				58.31	0.12	3				58.72	0.1	3			
10 µM PRMT2	T_{m1}			T_{m2}			T_{m1}			T_{m2}			T_{m1}			T_{m2}			T_{m1}			T_{m2}		
	55.22	55.25	55.17				56.51	56.18	56.57				57.33	56.64	56.9				58.5	58.5	58.48			
	Ave	SD	N	Ave	SD	N	Ave	SD	N	Ave	SD	N	Ave	SD	N	Ave	SD	N	Ave	SD	N	Ave	SD	N
	55.22	0.03	3				56.42	0.17	3				56.96	0.28	3				58.49	0.01	3			

Supporting Table 22. Tabulated data from densitometry analysis in Supporting Figure 16. This data was used to plot the bar charts shown in **Figures 4G-I.**

	Phosphor Signal (a.u.)			
**H3**				
[Histones] (mg/mL)	1:0 PRMT1:2	1:1 PRMT1:2	1:10 PRMT1:2	1:100 PRMT1:2
0.005	3430	3180	887	148
0.01	1970	1700	2210	100
0.025	1660	1830	3050	297
0.05	3340	5170	4870	811
0.075	4090	5680	4600	940
0.1	5530	5090	4560	1970
0.2	4360	4970	5900	2040
0.3	3100	4540	5060	1630
**H2A**				
[Histones] (mg/mL)	1:0 PRMT1:2	1:1 PRMT1:2	1:10 PRMT1:2	1:100 PRMT1:2
0.005	4190	4840	2360	1030
0.01	4310	4620	7010	1460
0.025	4860	3600	12200	2050
0.05	4200	4150	16200	4920
0.075	3700	6160	12200	7680
0.1	5260	2750	10200	15400
0.2	6160	4630	8260	15800
0.3	5650	1730	5190	13500
**H4**				
[Histones] (mg/mL)	1:0 PRMT1:2	1:1 PRMT1:2	1:10 PRMT1:2	1:100 PRMT1:2
0.005	1880	2960	1800	916
0.01	2540	3860	3800	847
0.025	4560	4990	6660	70
0.05	9540	12100	10200	807
0.075	9480	11700	15000	517
0.1	15000	11200	16200	11800
0.2	16900	14200	16200	13600
0.3	16300	11500	16400	16800

Supporting Table 23. Tabulated data from densitometry analysis Supporting Figure 16. This data was used for fitting purposes against linear, and nonlinear regression models to determine the kinetic parameters shown in **Table 1**.

**[H2A] (μM)**	**PRMT1/2 Ratio**											
	1:0			1:1			1:10			1:100		
	**Phosphor Signal (a.u.)**											
0	1367	987	1687	4410	4510	3110	7810	6810	8010	3587	3287	2887
0.1	1787	1167	1327	3410	3810	4110	8510	9810	8110	1097	1887	2487
0.5	2587	2487	1787	5410	5010	6710	10110	10310	11510	3187	5187	5487
1	2787	2087	3987	4810	4110	6710	10210	8710	8810	5487	5287	5187
5	2487	2987	3387	9910	11410	11410	12110	15210	14110	4787	5687	4887
10	9587	9287	8887	17510	21310	19910	21610	20010	18810	8487	8687	7787
15	7787	8287	9187	33710	38610	36310	28610	33810	31910	7087	6587	7987
20	14287	15787	13487	43610	46210	39810	48510	43810	43110	10487	10487	9287
25	20187	16387	18987	52310	52210	46810	44510	44710	37110	7287	6187	6987

Supporting Table 24. Tabulated data from densitometry analysis in Supporting Figure 17. This data was used for fitting purposes against linear, and nonlinear regression models to determine the kinetic parameters shown in **Table 1**.

**[H3] (μM)**	**PRMT1/2 Ratio**											
	**1:0**			1:1			1:10			1:100		
	**Phosphor Signal (a.u.)**											
0	1587	2127	1837	2150	1150	1940	1840	1490	2770	2467	2117	3607
0.1	2817	2697	2727	1120	1330	1750	3780	2730	3420	4207	4417	4527
0.5	3247	2957	3167	1810	1680	1740	3200	2370	2850	4267	4697	5017
1	5027	4967	5597	2160	240	760	6500	6100	2940	5297	4437	4227
5	10607	10907	11707	27300	31100	28200	12500	11500	11400	5007	4557	4487
10	14307	14607	13607	55200	58400	61400	34700	37300	31700	6907	6807	6707
15	8707	8707	8807	47900	43600	47600	32200	36600	42800	5267	5577	5937
20	10607	12607	12607	38500	37300	44000	44900	42200	40700	5887	6507	6307
25	9107	10007	9807	35100	40600	43400	51900	44400	57800	5357	6907	6067

Supporting Table 25. Tabulated data from densitometry analysis in Supporting Figure 18. This data was used for fitting purposes against linear, and nonlinear regression models to determine the kinetic parameters shown in **Table 1**.

**[H4] (μM)**	**PRMT1/2 Ratio**											
	**1:0**			**1:1**			**1:10**			**1:100**		
	**Phosphor Signal (a.u.)**											
0	4227	7607	3607	12607	15607	11907	19007	16607	10607	40800	38100	32400
0.1	6507	8007	8507	10807	12907	11807	23007	13407	15107	31000	31700	32400
0.5	5607	7607	5487	15907	19007	13407	22107	21607	22007	45400	46900	46000
1	14907	12307	11907	8907	11607	12107	13000	13300	12500	33100	39800	29100
5	19107	19807	17607	23907	24107	20407	23300	24100	23900	36700	38400	33200
10	27007	32507	30607	33207	27807	30807	34300	26900	30700	58500	57200	59400
15	43907	39707	38507	47007	48907	34507	34900	40700	45600	48100	47900	41000
20	46807	45207	54107	46207	35007	36007	38400	44100	36600	62400	60000	53900

Supporting Table 26. Relative densitometry analysis for Figure 5E. Data was normalized to the signal (Coomassie stain or phosphor image intensity) from the wildtype histone H2A reaction with PRMT1 using the band corresponding to histone H2A. The column on the far right shows the normalized ratios of phosphor image signal over the corresponding Coomassie stain signal.

Enzyme	H2A variant	% Coomassie	% Phosphor	Phosphor/Coomassie
	WT	100.00	100.00	1.00
	R3K	43.36	23.86	0.55
	R11K	61.28	84.26	1.38
PRMT1	R29N	109.40	109.64	1.00
	WT	126.17	140.61	1.11
	R3K	32.15	1.12	0.03
	R11K	69.80	59.39	0.85
PRMT1/2	R29N	84.56	117.26	1.39
